# X-ray scattering reveals ion clustering of dilute chromium species in molten chloride medium†

**DOI:** 10.1039/d1sc01224j

**Published:** 2021-05-19

**Authors:** Santanu Roy, Shobha Sharma, Waruni V. Karunaratne, Fei Wu, Ruchi Gakhar, Dmitry S. Maltsev, Phillip Halstenberg, Milinda Abeykoon, Simerjeet K. Gill, Yuanpeng Zhang, Shannon M. Mahurin, Sheng Dai, Vyacheslav S. Bryantsev, Claudio J. Margulis, Alexander S. Ivanov

**Affiliations:** Chemical Sciences Division, Oak Ridge National Laboratory P. O. Box 2008 Oak Ridge TN 37831 USA ivanova@ornl.gov bryantsevv@ornl.gov; Department of Chemistry, The University of Iowa IA 52242 USA claudio-margulis@uiowa.edu; Pyrochemistry and Molten Salt Systems Department, Idaho National Laboratory Idaho Falls ID 83415 USA; Department of Chemistry, University of Tennessee Knoxville TN 37996 USA; National Synchrotron Light Source II (NSLS-II), Brookhaven National Lab USA; Chemistry Division, Brookhaven National Lab Upton New York 11973 USA; Neutron Scattering Division, Oak Ridge National Laboratory Oak Ridge TN 37831 USA

## Abstract

Enhancing the solar energy storage and power delivery afforded by emerging molten salt-based technologies requires a fundamental understanding of the complex interplay between structure and dynamics of the ions in the high-temperature media. Here we report results from a comprehensive study integrating synchrotron X-ray scattering experiments, *ab initio* molecular dynamics simulations and rate theory concepts to investigate the behavior of dilute Cr^3+^ metal ions in a molten KCl–MgCl_2_ salt. Our analysis of experimental results assisted by a hybrid transition state-Marcus theory model reveals unexpected clustering of chromium species leading to the formation of persistent octahedral Cr–Cr dimers in the high-temperature low Cr^3+^ concentration melt. Furthermore, our integrated approach shows that dynamical processes in the molten salt system are primarily governed by the charge density of the constituent ions, with Cr^3+^ exhibiting the slowest short-time dynamics. These findings challenge several assumptions regarding specific ionic interactions and transport in molten salts, where aggregation of dilute species is not statistically expected, particularly at high temperature.

## Abstract 2

Ion clustering of dilute chromium species was unexpectedly revealed in a high-temperature molten chloride salt, challenging several long-held assumptions regarding specific ionic interactions and transport in molten ionic media.

## Graphical abstract

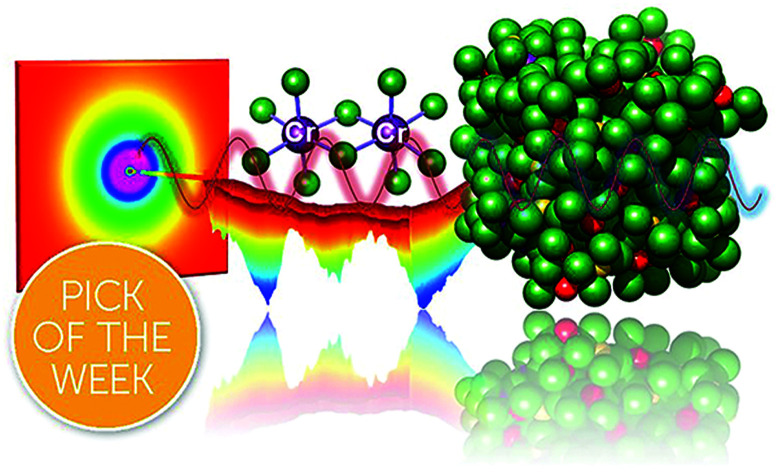

## Introduction

Simple inorganic molten salts have emerged as important media for the development of modern concentrating solar power (CSP) plants and next-generation molten salt nuclear reactors (MSRs) having significant potential to provide sustainable, carbon-free energy for the future.^1–4^ Among different salt formulations, mixtures of potassium chloride (KCl) and magnesium chloride (MgCl_2_) hold special promise for these technologies, owing to their stability under high-temperature regimes and their exceptional thermal characteristics.^5–9^ In addition, chloride salts are abundant and therefore relatively inexpensive. However, they can be very corrosive to metal alloy materials under the extreme, real-world operating conditions (873–1073 K) of CSP and MSR systems.^10–12^ A major issue is related to chromium being primarily depleted from the alloys upon contact with the molten KCl–MgCl_2_, leading to the formation of dilute CrCl_3_ in the melt.^10^

Despite extensive investigations of molten salts in the past, the only insights regarding how low-concentration Cr^3+^ metal ions behave in the high-temperature ionic medium have been achieved through optical spectroscopy.^13,14^ The absence of long-range order, such as that present in a single crystal or powder sample, limits the usefulness of conventional in-lab X-ray diffraction equipment using low-photon-energy sources, while the low metal content limits the information available from Raman spectroscopy (ESI Fig. 1†). X-ray absorption spectroscopy (XAS) is a powerful tool to investigate metal ion speciation,^15–18^ but provides reliable information only about the first coordination sphere, and recent XAS experiments were unable to determine the local structure of Cr^3+^ in a KCl–MgCl_2_ molten salt mixture at the high temperatures (>973 K)^19^ that are relevant to technological processes in CSP and MSR applications. The accurate identification and understanding of coordination environments and dynamic structural behaviour of chromium species could provide useful insights into the processes of corrosion and transport in molten salts. At a more fundamental level, this knowledge will broaden our general understanding of metal ion solvation in unconventional ionic media at high temperature.

In this study, we demonstrate a highly integrated approach combining theory, simulations and high-energy synchrotron X-ray scattering experiments to elucidate the atomic-scale structure and dynamics of dilute Cr^3+^ species in the KCl–MgCl_2_ molten salt at 1073 K. In order to enhance the information about the local structure of Cr^3+^ in a matrix of strong X-ray scattering ions, we applied a differential pair distribution function (dPDF) method. This technique used in conjunction with *ab initio* molecular dynamics (AIMD) simulations, reverse Monte Carlo (RMC) modeling and theory enabled us to interpret and reveal information about local Cr^3+^ environments. While the dPDF technique has been used to investigate uranyl complexes^20^ and CO_2_ (ref. 21) in aqueous solutions as well as solvation shells around various nanoparticles,^22–24^ to the best of our knowledge, this is the first instance of its use for metal ions dissolved in other highly ionic media under extreme temperature conditions. In addition, to understand the local dynamics of Cr^3+^ as compared to that of other ions in such complex medium, we have formulated and used a hybrid transition state (TS)-Marcus theory approach. Using this rate theory model, we found that dynamical processes in the melt are highly sensitive to the charge density of the constituent ions, with Cr^3+^ showing the slowest short-time dynamics. Perhaps surprisingly for such a high-temperature low-Cr^3+^ concentration system, our studies found that octahedrally coordinated Cr^3+^ ions tend to dimerize, forming persistent structures.

## Results and discussion

**Fig. 1:**
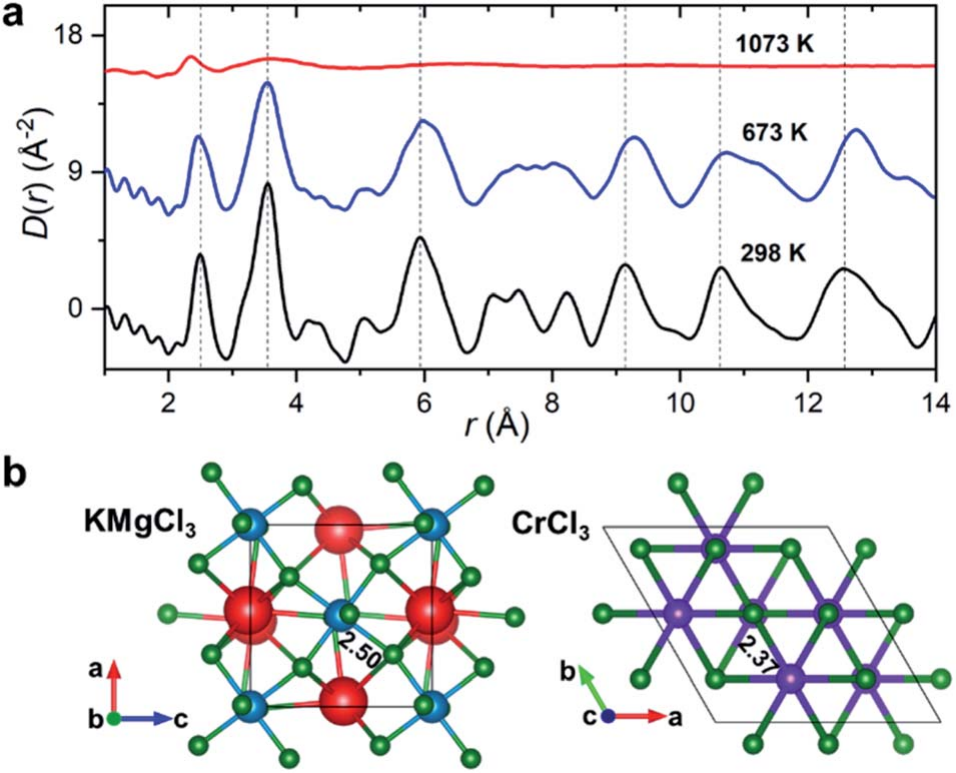
Temperature-dependent PDF results. (a) X-ray reduced pair distribution functions, *D*(*r*), for the KCl–MgCl_2_ salt mixture (50 : 50 mol%). (b) KMgCl_3_ (*Pnma*) and CrCl_3_ (*P*3_1_12) crystal structures with highlighted Mg–Cl and Cr–Cl average bond lengths in Å. Color scheme: K, red; Mg, blue; Cr, purple; Cl, green. Vertical dashed lines in (a) are guides to the eye.

Synchrotron X-ray diffraction measurements were initially performed on the salt mixture of 50 : 50 mol% KCl–MgCl_2_. To allow for the observation of structural changes with the increase of temperature, the data were collected at 298 K, 673 K (below the melting point), and 1073 K (molten state) using a customized furnace (ESI Fig. 2†) specifically designed to hold samples in quartz capillaries at high temperature.^25^ Our measured coherent scattering intensities provide the X-ray structure factor, *S*(*Q*), where the momentum transfer, *Q*, is 4π sin(*θ*)/*λ* (2*θ* is the scattering angle and *λ* is the incident X-ray wavelength). The Fourier transform of *S*(*Q*) gives pair distribution functions [*G*(*r*) or *D*(*r*) = 4π*ρ*_0_*rG*(*r*), where *ρ*_0_ is the ionic number density] that are related to the probability of finding ion pairs with a given separation distance *r*^26,27^ (see ESI† for *S*(*Q*), *G*(*r*) and *D*(*r*) definitions). Fig. 1a shows the evolution of *D*(*r*) as a function of temperature. Consistent with the increasing disorder and the loss of crystalline symmetries in the solid salt above the melting point (∼760 K), features in *D*(*r*) broaden upon heating.

Based on the relative X-ray weighting factors (ESI Fig. 3†) and the available orthorhombic KMgCl_3_ crystal structure (Fig. 1b) corresponding to the room temperature phase of KCl–MgCl_2_, the first peak in the *D*(*r*)s is primarily dominated by short-range Mg–Cl ionic correlations. The experimentally determined Mg–Cl average bond length contracts on heating from 2.50 Å at 298 K to 2.47 Å at 673 K, achieving the shortest distance of 2.37 Å in the molten state, whereas longer-range order correlations show the opposite trend consistent with thermal expansion (Fig. 1a), and at 1073 K they significantly broaden or disappear as expected in the molten state. What may at first seem counterintuitive, the observed shrinking of the cation–anion close-contact distances is quite typical for inorganic salts and is associated with a decrease in the coordination numbers on melting, often leading to shorter nearest-neighbour bond lengths than those observed in the solid state.^25,28–30^ The characteristically broad peaks in *D*(*r*) at the highest temperature associated with the liquid state make the interpretation of scattering results difficult, requiring more sophisticated procedures in the data analysis.

**Fig. 2:**
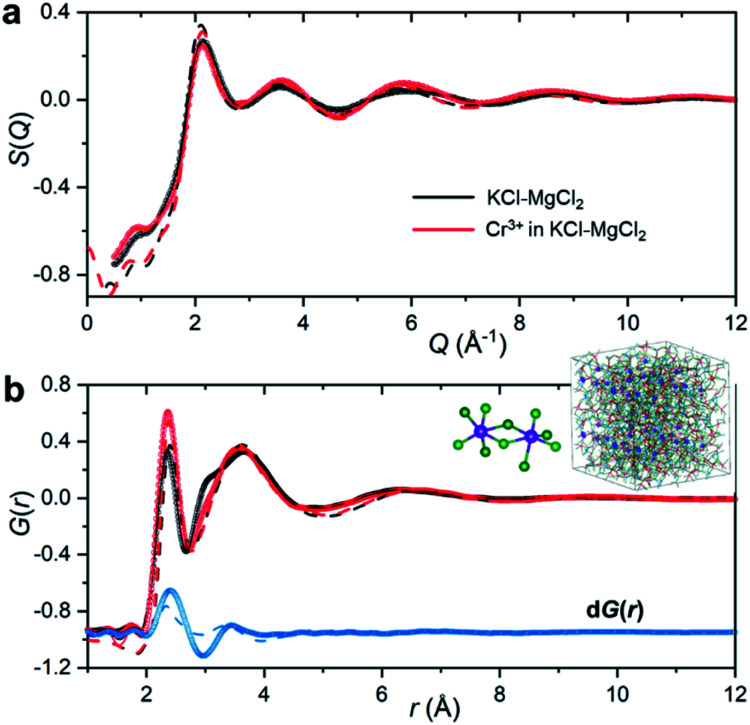
X-ray scattering measurements of the KCl–MgCl_2_ (black) and CrCl_3_–KCl–MgCl_2_ (red) molten salt mixtures at 1073 K. (a) X-ray structure functions *S*(*Q*). (b) X-ray pair distribution functions *G*(*r*) generated from the *S*(*Q*) patterns in (a), and the corresponding differential PDF d*G*(*r*) (blue), which is offset for clarity. Circles represent the experimental measurements; solid lines are from the RMC modeling (due to a nearly perfect match the discrepancy between the measured datapoints and the RMC model is smaller than the line thickness); dashed lines are from the AIMD simulations. The inset shows a snapshot from the RMC modeling highlighting ionic clustering of Cr^3+^ species (enlarged purple spheres) and representative chromium chloride dimers present in the melt.

To emulate the presence of dilute metal ion species in the molten ionic medium, we added a small amount of CrCl_3_ (5 mol%) to the KCl–MgCl_2_ salt mixture. Because the concentration of Cr^3+^ in the sample is low, information in *S*(*Q*) and the total PDF about its interactions with other species in the matrix will be masked by the KCl–MgCl_2_ solvent. This problem is compounded by the fact that interionic distances between Cr^3+^ and other species can be quite similar to those between solvent species. Therefore, in order to capture the small variation in *S*(*Q*) and *G*(*r*) caused by the presence of dilute Cr^3+^ species, we performed high-energy X-ray diffraction measurements for the molten salt mixture before and after addition of CrCl_3_ (Fig. 2).

As expected, the obtained *S*(*Q*)s and *G*(*r*)s show very subtle differences for the CrCl_3_-loaded and pristine salt systems (Fig. 2). Close examination reveals an enhancement of the first peak intensity at ∼2.37 Å, suggesting that Cr–Cl first neighbor distances nearly coincide with those of Mg–Cl in the melt. These results also imply that Cr^3+^ is surrounded by an octahedral configuration of Cl^−^ anions, since the Cr–Cl bond lengths do not seem to change in the molten state as compared to the crystalline form of octahedral CrCl_3_ in Fig. 1b. This evidence is further supported by our high-temperature optical absorption spectroscopy results (ESI Fig. 4†). The absorption spectrum consists of two bands in the visible region that correspond to the ^4^A_2_ → ^4^T_2_ (^4^F) and ^4^A_2_ → ^4^T_1_ (^4^F) transitions for the peaks at 12 000 cm^−1^ and 18 000 cm^−1^, respectively, as well as a charge transfer band corresponding to the ^4^A_2_ → ^4^T_1_ (^4^P) transition, consistent with the spectrum of dilute Cr^3+^ in the LiCl–KCl eutectic melt where octahedral coordination was previously identified.^14^

The differential PDF, d*G*(*r*), obtained by direct subtraction of the reference PDF of the KCl–MgCl_2_ molten salt from that of the CrCl_3_-loaded mixture is shown in Fig. 2b. We performed AIMD simulations to interpret the differential PDF results. Fig. 2a shows very good agreement between the experimental and simulated *S*(*Q*) beyond 2 Å^−1^. At lower *Q* values, a good match is not expected due to the limited size of our AIMD boxes. RMC simulations, starting from the AIMD snapshot configuration where the box size has been quadrupled, completely resolved this discrepancy, providing a perfect match between the experiment and the RMC model (Fig. 2). The comparison of radial distribution functions, *g*(*r*), in ESI Fig. 5† indicates a good agreement between the results from RMC and AIMD simulations, despite the limitation of the latter technique to reproduce the *S*(*Q*) features at low *Q*. This provides some confidence in the analysis of the dPDF at short distances based on the first principles results. In addition, close agreement of the AIMD simulations with the experimental X-ray *G*(*r*) allows full isolation of all ionic correlations (Cr–Cl, Cr–Cr, Cr–Mg, Cr–K, *etc.*) *via* the Fourier transformation of the partial subcomponents of *S*(*Q*) (ESI Fig. 6†). This can also provide valuable information as to which pair interactions contribute to the differential PDF at specific distances (ESI Fig. 7†). It is worth mentioning, however, that while computational *G*(*r*) functions reproduce the experiment well, the match in the differential PDF is apparently worse, highlighting the complexity of extracting data from the differential technique, which makes use of small changes in the overall signal upon introduction of dilute Cr^3+^ species.

**Fig. 3:**
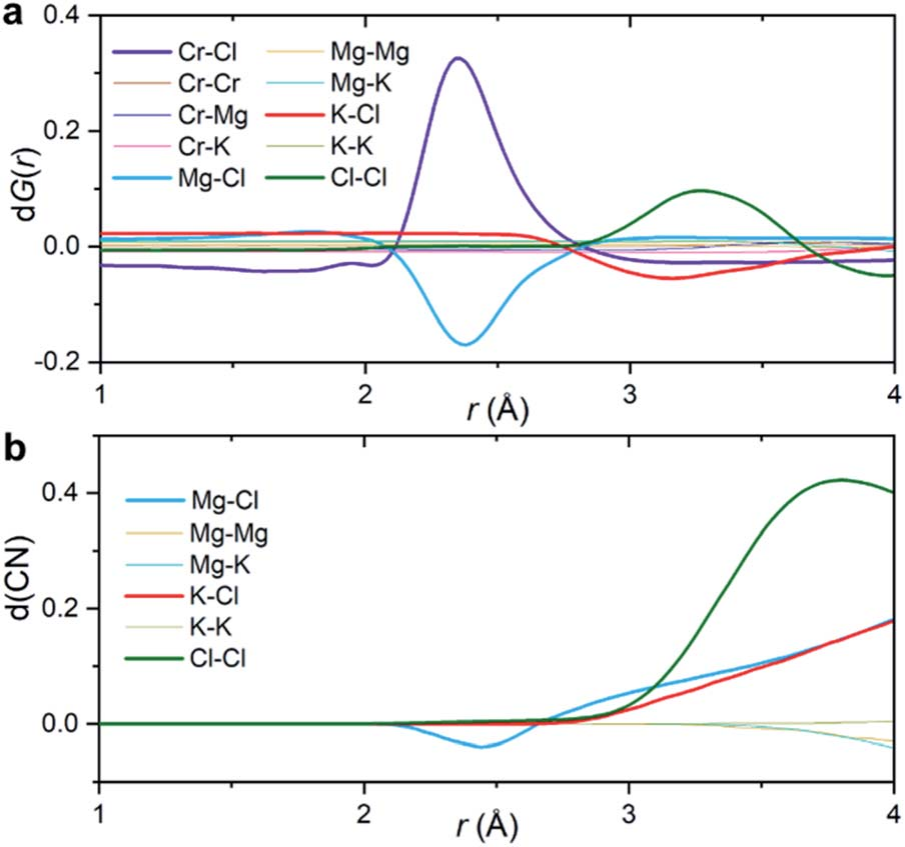
Interpretation of the differential PDF. (a) Partial subcomponents of d*G*(*r*) from AIMD simulations. (b) Differential coordination number d(CN) for the solvent–solvent correlations, showing the difference between the cumulative coordination number in the CrCl_3_ loaded system and the pristine KCl–MgCl_2_ molten salt.

Fig. 3a shows the partial subcomponents of d*G*(*r*) as defined from the subtraction of partial subcomponents from the two samples in the first principles simulations. One may see that the first and most prominent peak in d*G*(*r*) mainly results from two competing contributions, a positive peak from Cr–Cl and a negative peak at similar distances from Mg–Cl correlations. This positive peak is very simple to interpret: in a differential PDF calculation, terms that only exist in the CrCl_3_-loaded sample are equal to the corresponding partial subcomponent of *G*(*r*), *i.e.* d*G*_Cr–Cl_(*r*) = *G*_Cr–Cl_(*r*). In other words, the first peak in d*G*(*r*) shows the location of the closest contact between Cr^3+^ and Cl^−^.

Instead, solvent–solvent type terms that appear in both systems, and are of the same order of magnitude, require more nuance in our analysis and interpretation. Fig. 3b displays the differential coordination number, d(CN), of selected ions. A positive d(CN) means that the cumulative sum of ions of certain class up to a distance *r* surrounding a central ion is larger in the Cr^3+^-containing sample, whereas a negative differential coordination number means that this quantity is smaller. We see for example, that whereas the depletion in the case of d*G*_Mg–Cl_(*r*) below 2.5 Å is consistent with a depletion in the coordination number up to that distance (and also with a depletion in the actual computed radial distribution function *g*_Mg–Cl_(*r*) at that distance (ESI Fig. 8†)), this is not the case for the K–Cl correlation at ∼3 Å. Here, consistent with the behavior of d*G*_K–Cl_(*r*), the first peak in *g*(*r*) for this ionic pair is slightly lower for the system with CrCl_3_, but the coordination number of Cl^−^ around K^+^ is larger (Fig. 3b). In other words, a negative/positive peak in the partial subcomponents of d*G*(*r*) for solvent–solvent components, cannot be simply interpreted as a depletion/increase in coordination. This is because the addition of a solute (CrCl_3_) results in a concomitant change in the molar volume and hence in the number density of the different species. This phenomenon is particularly complicated in molten salts where a solute, in our case Cr^3+^, necessarily comes combined with Cl^−^ counter ions that are both part of the solute and the solvent. Instead, the experimental observable d*G*(*r*) depends on mole fractions of the ions and their effective number of electrons.

The second peak in d*G*(*r*) is dominated by Cl–Cl interactions between 3 and 3.5 Å. In this case, the enhancement is both in d*G*_Cl–Cl_(*r*) and the coordination number (Fig. 3). It mostly represents a shift in the *g*_Cl–Cl_(*r*) towards shorter distances (ESI Fig. 8†) due to the strong Cr–Cl attractive interactions when dilute Cr^3+^ ions are present in the melt. This behavior is consistent with the Cl–Cl contraction observed recently in neutron diffraction experiments for the binary CrCl_3_–NaCl melt with much higher CrCl_3_ content (22 mol%).^31^

**Fig. 4:**
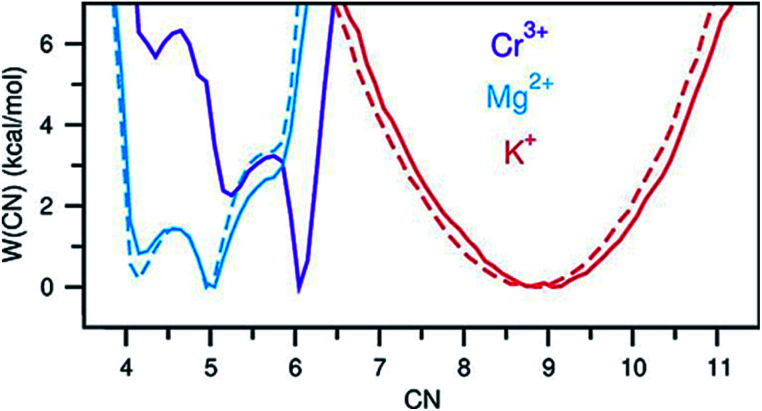
Free energy as a function of coordination number of Cr^3+^, Mg^2+^, and K^+^. Solid and dashed lines indicate the KCl–MgCl_2_ salt mixtures with and without Cr^3+^, respectively.

A perhaps unexpected result from our PDF fitting using RMC is that Cr^3+^ ions tend to aggregate, mainly forming [Cr_2_Cl_10_]^4−^ dimeric species in the KCl–MgCl_2_ molten salt even at low concentration and high temperature (see inset in Fig. 2b). Our AIMD simulations, starting from the initial configuration where Cr^3+^ ions were deliberately placed far away from each other, confirm Cr^3+^ clustering after about 10 ps. To gain additional insight into the observed ion clustering and coordination environments, we explored free energy landscapes that describe the distribution and relative stability of various coordination structures in the melt. As can be seen from the free energy profiles in Fig. 4, Cr^3+^ can be surrounded by 4–6 chloride anions in the first solvation shell, with the octahedral coordination clearly being the most favourable. The 5- and 4-coordinated states are rarely accessible due to rather high free energy barriers. This is consistent with our optical spectroscopy measurements, showing the persistence of spectral features and hence the octahedral geometry of the Cr^3+^ species over a wide range of temperatures (ESI Fig. 4†). In contrast, the 5- and 4-coordinated states of the solvent Mg^2+^ ions are almost equally probable. The free energy barrier between them is less than 1 kcal mol^−1^, which is well below the thermal energy at 1073 K (2.13 kcal mol^−1^) affording frequent interconversions of these states. Unlike highly charged Cr^3+^ and Mg^2+^, K^+^ ions exhibit a single 9-coordinated state, *i.e.* 9 Cl^−^ around a K^+^ ion (Fig. 4). Addition of Cr^3+^ ions slightly modifies the free energy profiles for the solvent cations. The 4-coordinated state becomes less stable for Mg^2+^, indicating an increase in the average coordination number. This is in agreement with our results in Fig. 3b, showing positive d(CN) values for Mg–Cl at the cutoff distance (3.5 Å) which we use to define the average coordination number for Mg^2+^. The free energy profile for K^+^ shifts slightly to a higher coordination number as well (Fig. 4), consistent with the corresponding d(CN) for K–Cl. This can be likely attributed to the high charge density of Cr^3+^ capable of polarizing Cl^−^ significantly, which results in weaker Cl–Cl repulsive interactions and thus allows more Cl^−^ ions in the first coordination shell of the cations.

**Fig. 5:**
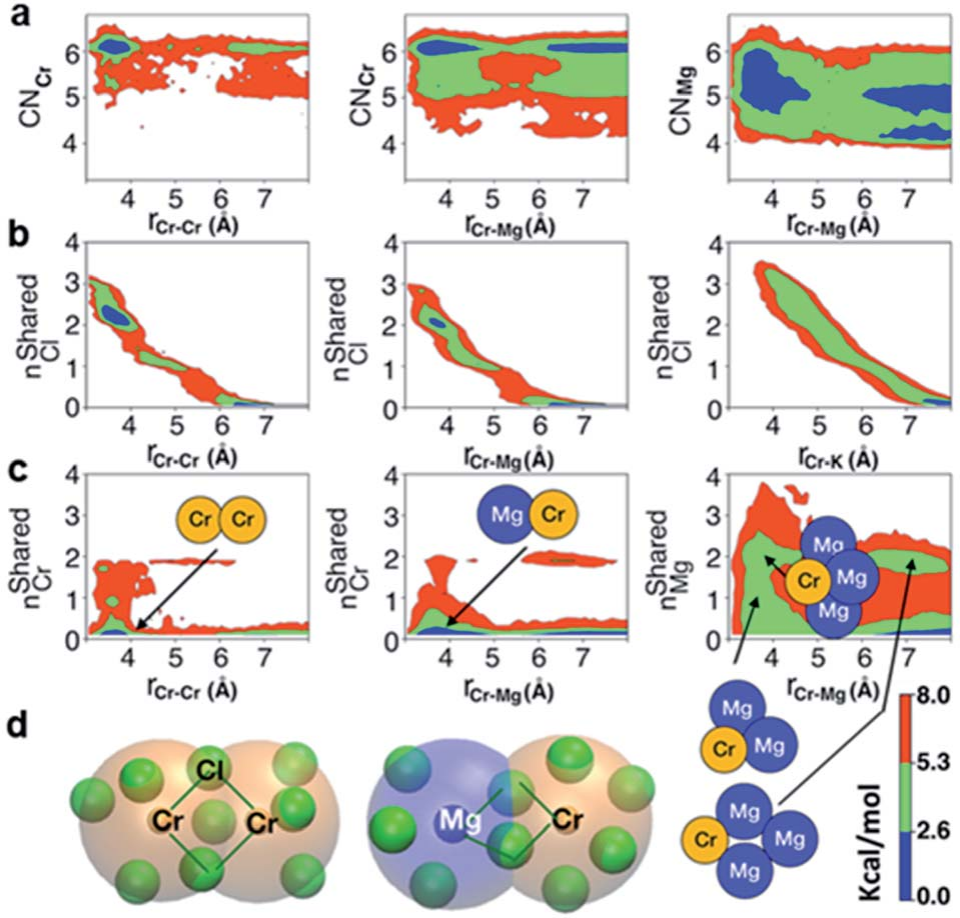
2D-free energy surfaces highlighting (a) most probable coordination number of Cr^3+^ (CN_Cr_) and Mg^2+^ (CN_Mg_) across a range of Cr–Cr and Cr–Mg distances; (b) the chloride-shared and chloride-separated states for Cr–Cr, Cr–Mg, and Cr–K dimeric configurations; (c) number of Cr^3+^ ions coordinating with different Cr^3+^ and Mg^2+^ ions, and number of Mg^2+^ ions coordinating with Cr^3+^ and different Mg^2+^ ions *via* chlorides, with the respective dimers, trimers, and tetramers pointing to the region where they form; (d) representative chloride-shared [Cr_2_Cl_10_]^4−^ and [CrMgCl_9_]^4−^ dimeric configurations present in the CrCl_3_–KCl–MgCl_2_ melt. Color bar represents the contour levels in the 2D-free energy surfaces.

The polarization effect caused by Cr^3+^ ions is better understood by analyzing the free energy surfaces for the chloride shared cation–cation configurations. The 2D-free energy surfaces in Fig. 5a show that the coordination environment of Cr^3+^ remains octahedral regardless of whether it makes close contact with different Cr^3+^ or Mg^2+^ ions. However, Mg^2+^ adopts a slightly higher coordination number (between 5 and 6) when contacting with Cr^3+^, while switching back and forth between the 4- and 5-coordinated states as it moves away from the highly charged Cr^3+^. In other words, Mg^2+^ draws more Cl^−^ ions in its first solvation shell when forming a dimeric species with Cr^3+^. The free energy surfaces in Fig. 5b demonstrate that Cr^3+^ and Mg^2+^ ions in their most probable [Cr_2_Cl_10_]^4−^ and [CrMgCl_9_]^4−^ dimeric configurations (Fig. 5d) are linked together through two bridging Cl^−^ anions. In addition, the existence of chloride-shared dimeric Cr–K configurations is rare according to our calculations (Fig. 5b), hence stable dimers can only be formed between multivalent cations.

One may expect that the high charge density of Cr^3+^ can also induce the formation of more complex aggregates, such as trimers or even longer chains. However, only the Cr–Cr dimeric species were found in the melt, likely due to the low concentration of Cr^3+^ and high temperature of the system. The 2D-free energy surface in Fig. 5c (left panel) justifies this, showing that even the formation of Cr–Cr–Cr trimers is highly improbable. The same conclusion can be drawn for Cr–Mg–Cr trimers, which are unlikely to exist (Fig. 5c, middle panel). However, free energetically metastable Cr–Mg–Mg trimers and Cr–Mg–Mg–Mg tetramers can still form (Fig. 5c, right panel) due to the relatively high concentration of Mg^2+^ ions in the KCl–MgCl_2_ molten salt. Whereas this analysis can only reveal correlations that are shorter-range, based on our experience studying KCl–MgCl_2_ mixtures,^25^ it is likely that observations in Fig. 5c (right panel) imply that dilute Cr^3+^ is becoming a component in Mg^2+^ networks.

**Fig. 6:**
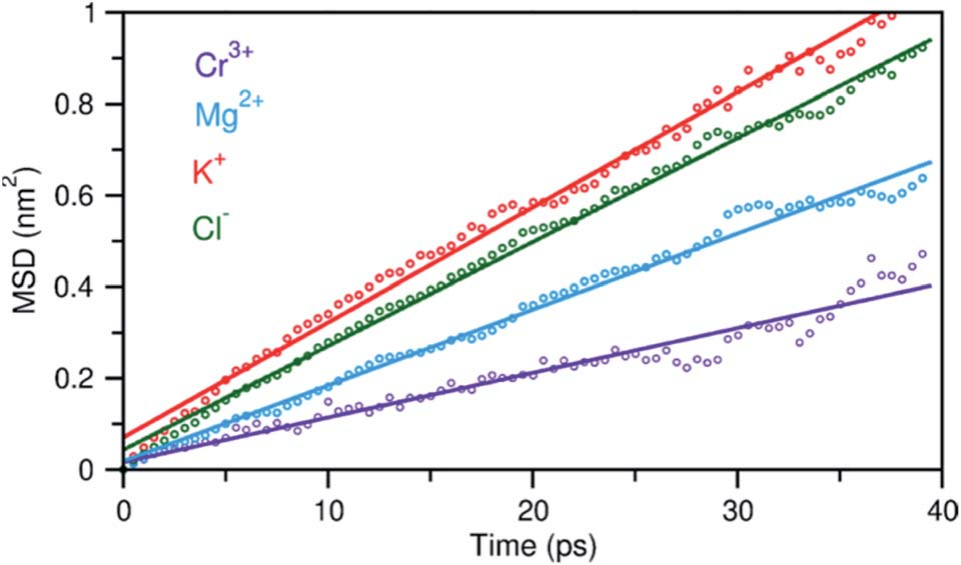
Mean square displacement (MSD) of ions as a function of time, indicating faster dynamics in the order: K^+^ > Cl^−^ > Mg^2+^ > Cr^3+^. Dots represent the MSD data and solid lines are linear fits.

In addition to the structural diversity, the dynamics of ions with different charge density can be highly heterogeneous in the multicomponent molten salt system. To examine this, we analyzed the mean square displacement (MSD) of each ion over time. Fig. 6 shows that the large ions with low charge density, K^+^ and Cl^−^, move at faster rates than the smaller ions with high charge density, Mg^2+^ and Cr^3+^. Typically, the MSD analysis can provide diffusion coefficients, but it requires information about long-time displacements that is lacking in our 40 ps AIMD simulation time. Nevertheless, the short-time dynamics of these ions are still critically useful and can be correlated with ion exchange and clustering kinetics.^32,33^ We therefore study these processes around the solute Cr^3+^, the solvent Mg^2+^ and K^+^ cations using a hybrid TS-Marcus theory framework, which allows us to elucidate the origin of heterogeneous local dynamics in our multicomponent ionic system.

Conventional rate theory-based studies of ion pairing and solvation typically utilized the interionic/intermolecular distance as the reaction coordinate to determine the transition rates between the reactant and product states.^34–37^ However, recent studies indicate that inclusion of a second reaction coordinate associated with solvent fluctuations is necessary to provide a better understanding of the underlying mechanisms and rates.^38–41^ These studies show that the fluctuating coordination number or the electric field are both reasonable collective variables that can be used to represent solvent fluctuations. We find the electric field on a solute to be a better reaction coordinate because it is determined by considering the contribution from the entire solvent, whereas the coordination number only accounts for the local solvent environment. Herein, considering that the reactant and product states can be resolved as well-separated states in the electric field space, we formulate a hybrid TS-Marcus framework (see ESI† for more details) to determine the reactant-to-product transition rates describing ion exchange and clustering processes.

**Fig. 7:**
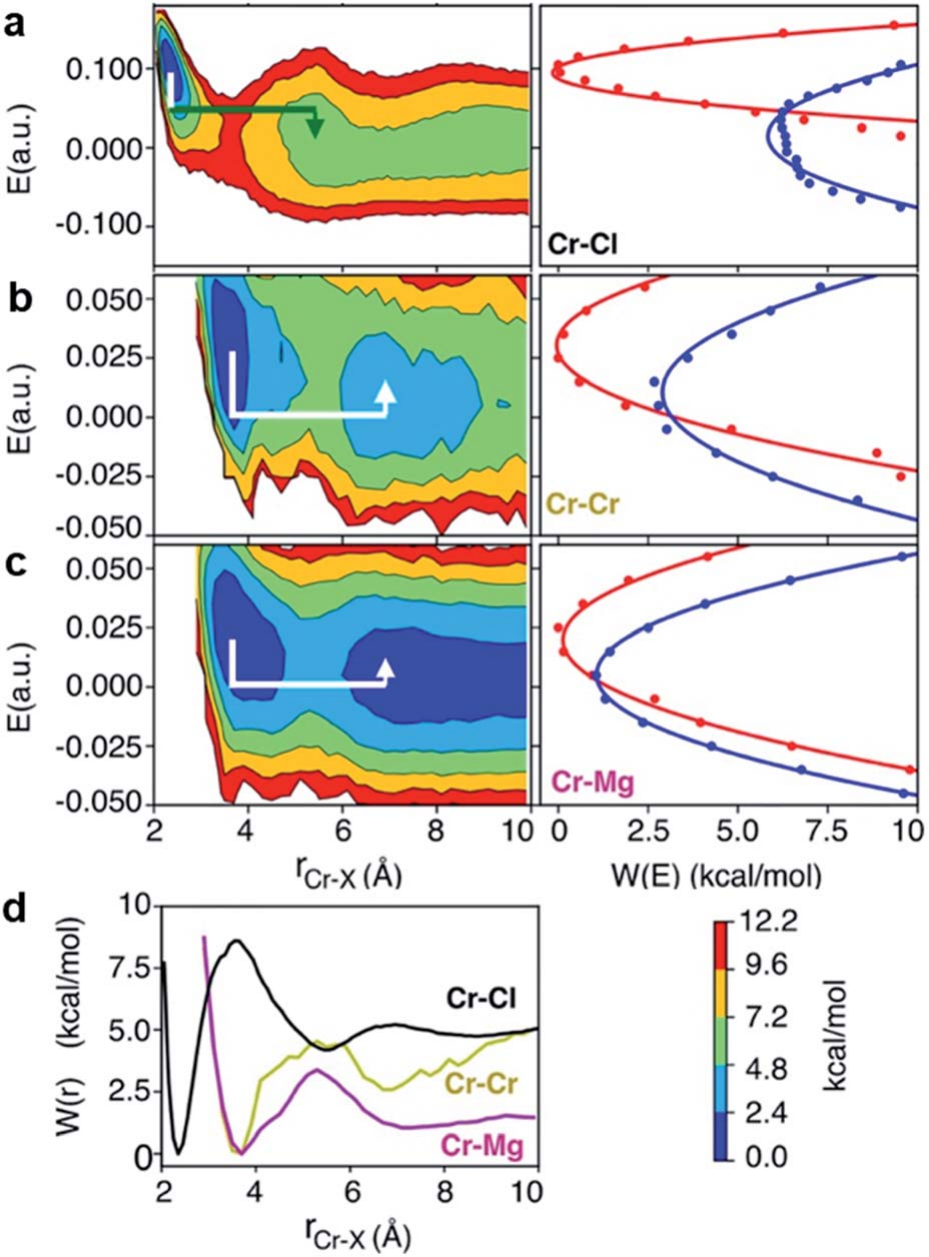
2D free energy surfaces, involving Cr–Cl, Cr–Cr, Cr–Mg distance and electric field, *E*, experienced by (a) Cl, (b) Cr, and (c) Mg with corresponding Marcus parabolas (solid lines are parabolic fits and dots are actual data), describing chloride exchange around Cr^3+^ and dissociation of the Cr–Cr and Cr–Mg dimers. The lines with arrows are the Marcus pathways showing how reorganization of ionic media causes electric field rearrangement and drives the exchange and dissociation events. (d) The corresponding 1D free energy profiles.

**Fig. 8:**
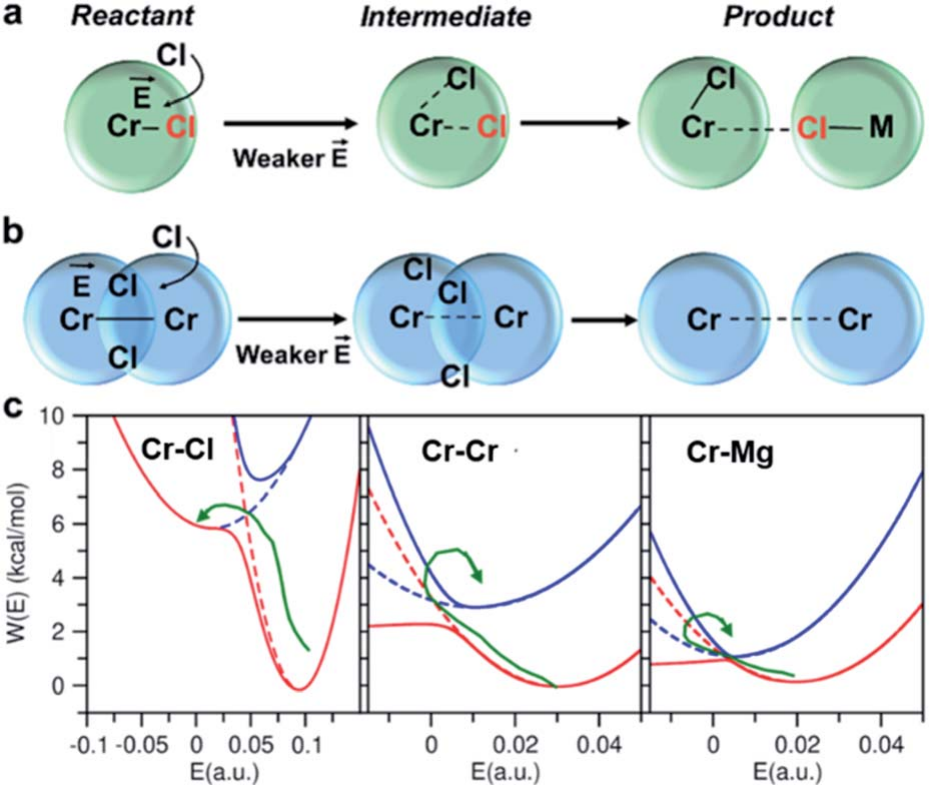
Reactant, intermediate, and product states for (a) chloride exchange and (b) dissociation of dimeric complexes, indicating that the solvent rearrangement-driven motion of Cl^−^ into the solvation shell changes the local electrostatic environment, triggering the exchange/dissociation process. This change in the electrostatic environment is gleaned from (c) the lower and higher adiabatic energy surfaces (solid lines) obtained from coupled Marcus diabats (dots) in the electric field space. The reactant-to-product transition path is shown by a green arrow.

To investigate local dynamics around Cr^3+^, we focused on three specific processes: chloride exchange around Cr^3+^ and the dissociation of Cr–Cr and Cr–Mg dimers. For the case of chloride exchange, we obtained the free energy surface as a function of the Cr–Cl distance and the electric field that Cl^−^ experiences along the 
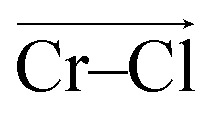
 direction (Fig. 7a). Analogously for the cases of Cr–Cr (Fig. 7b) or Cr–Mg (Fig. 7c) dissociation, the free energy is for the Cr–Cr or Cr–Mg distance and the electric field that the Cr^3+^ or Mg^2+^ ion experiences along the 
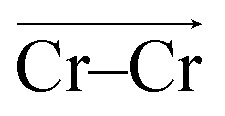
 or 
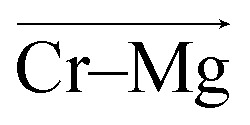
 direction. When Cl^−^ is located within the first coordination shell of Cr^3+^ representing the reactant state of the chloride exchange process (Fig. 8a), the Cl^−^ ion experiences a strong field. However, as soon as another Cl^−^ moves from the bulk solution into the Cr^3+^ solvation shell driven by solvent rearrangement, the local electrostatic environment changes in a way to reduce the positive field experienced by the chromium bound Cl^−^ ion (the intermediate state in Fig. 8a). Further weakening of the Cr–Cl interaction leads to the transition toward the product equilibrium, in which the initially bound Cl^−^ completely escaped the first coordination sphere of Cr^3+^. In the product state, the Cl^−^ ion experiences a broad range of negative and positive fields distributed around the mean value of ∼0. This is due to the possibility of Cl^−^ interacting with all other ions in an isotropic environment.

The discussed pathway of chloride exchange is depicted in Fig. 7a using an arrow on the free energy surface. For the dissociation of Cr–Cr and Cr–Mg dimers, the underlying mechanism can be explained using a similar approach as illustrated in Fig. 7b, c and 8b. In these cases, the field in the reactant state is already week due to sharing Cl^−^ ions between the two cations in the dimeric configurations, leading to the partial screening of the Cr–Cr and Cr–Mg interactions. After a full separation in the product state, their interaction strength vanishes completely.

It is worth noting that the minima representing the equilibrium close-contact (*r*_R_) and dissociated (*r*_P_) distances on the 2D-surfaces are identical to those on the 1D-free energy profiles along the interionic distance (Fig. 7d). To perform a Marcus theory-based analysis, we curved out a couple of slices from the 2D-free energy surfaces along *r* = *r*_R_ and *r* = *r*_P_ resulting in the reactant and product diabats in the electric field space, which we modeled as parabolas (Fig. 7 and ESI Table 1†). The coupling between these diabats generates lower and higher adiabatic free energy surfaces shown in Fig. 8c. From the perspective of Marcus theory, we find that while chloride exchange around Cr^3+^ can be described as the reactant (strong field)-to-product (near zero field) transition through the crossing region (identified as “normal region”) on the lower adiabatic surface, a nonadiabatic jump from the lower to the higher adiabatic surface through the crossing region (identified as “inverted region”) is required to drive the dissociation of the Cr–Cr or Cr–Mg dimeric structures. The crossing region corresponds to the respective intermediate configuration depicted in Fig. 8a and b, where the field gets weakened, subsequently triggering the transition to the product state.

To discern between the local solvent dynamics around the Cr^3+^ ion and the overall solvent dynamics in the molten salt system, we explored 2D-free energy surfaces and their respective Marcus diabatic and adiabatic states, and examined chloride exchange around solvent Mg^2+^ and K^+^ ions as well as dissociation of the Mg–Mg dimers (ESI Fig. 9 and 10†). While chloride exchange around Mg^2+^ is an adiabatic process, chloride exchange around K^+^ and Mg–Mg dissociation are nonadiabatic processes. Considering all the processes involving both solute and solvent ions, we find a trend that when an ion experiences a strong field in a close-contact configuration, the reactant and the product states are well-separated in the electric field space and cross at the “normal region”, exhibiting adiabatic interconversion. On the other hand, a weak field on an ion at a close-contact configuration does not separate the reactant and product states well and is responsible for these states crossing at the “inverted” region. Thus, the nature of these dynamical processes is highly sensitive to the charge density of ions in the molten salt.

There are several different factors that govern the kinetics of chloride exchange and dissociation of the dimers at a given temperature. As discussed in detail in the ESI,† the reactant-to-product transition rate (given by eqn (1)) depends on the free energy barrier (*W*_tot_), the transmission coefficients (*κ*_LZ_) representing barrier recrossing events, and the mass-weighted “reactant volume” 
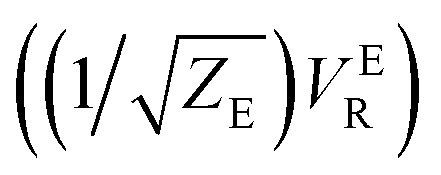
 at the given thermal energy, 1/*β*.1
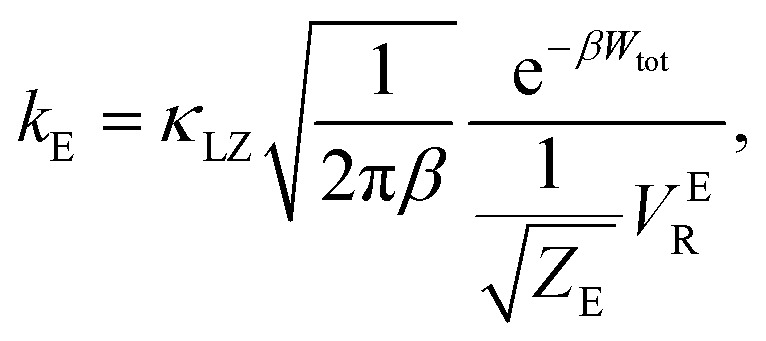


Of all these parameters, we find that the most important ones are the free energy barriers and transmission coefficients that change much more drastically than the mass-weighted “reactant volume” as we go across different cases (ESI Table 1†). The trend in the total barriers is consistent with the strength of interionic electrostatics in the close-contact reactant state. For instance, chloride exchange around Cr^3+^ has the highest barrier of ∼10 kcal mol^−1^, while Mg–Mg dissociation has to overcome a very low barrier of ∼2 kcal mol^−1^. Because of the difference in barrier heights, if we consider no barrier recrossing chloride exchange around Cr^3+^ can be slower than for Cr–Cr and Cr–Mg dissociation (for no or minimal barrier-recrossing, *κ*_LZ_ ∼ 1, while *κ*_LZ_ ≪ 1 for a significantly large number of recrossing events). However, when barrier recrossing is taken into account, Cr–Cr separation and chloride exchange around Cr^3+^ appear to occur on the same timescale of about 32–35 ps. This is because of the low transmission coefficient (0.116) for the separation of Cr–Cr dimers. This low *κ*_LZ_ implies that dissociation of these species is normally promptly followed by recombination. This inseparability of the time scales associated with chloride exchange around Cr^3+^ and the Cr–Cr dissociation implies that dilute chromium species may exist in the melt as kinetically stable [Cr_2_Cl_10_]^4−^ clusters for several tens of picoseconds. In contrast, the lifetime of the Cr–Mg dimers is much shorter (∼4 ps) than the timescales for chloride exchange around Cr^3+^ and Mg^2+^ ions. This is because of the much smaller barrier and larger transmission coefficient. The dynamics of the solvent ions follow a similar trend, with the barrier height making chloride exchange around Mg^2+^ slower than chloride exchange around K^+^ and Mg–Mg dissociation. In fact, without considering barrier recrossing, the latter two processes exhibit the fastest dynamics in the molten salt system at the same timescale of 0.5 ps. Interestingly, the transmission coefficient for Mg–Mg dissociation is extremely small (*κ*_LZ_ ∼ 0.009), suggesting that rapid dissociation of Mg–Mg pairs is immediately followed by recombination providing dynamical stability of the –Mg–Mg– chains.

## Conclusions

The dynamic structural behaviour of dilute Cr^3+^ species in a high-temperature ionic chloride medium was investigated by means of high-energy X-ray scattering and AIMD simulations. In the molten KCl–MgCl_2_ mixture, the first solvation shell of Cr^3+^ comprises 6 Cl^−^ anions, with computational results indicating persistence of the octahedral environment at least up to 1073 K. Therefore, the six-fold coordination of Cr^3+^, typically observed in the solid state, also occurs in the liquid chloride mixture, which can be explained by the high crystal field stabilization energy for the octahedral Cr^3+^ complex.^42^ Beyond simply identifying the Cr^3+^ coordination environment, our findings challenge the paradigm of molten salts being a “sea of cations in a sea of anions,” where aggregation of dilute species is not expected due to high temperature conditions. Instead, the results of our studies indicate the formation of dinuclear chloride-shared Cr–Cr clusters with a relatively long lifetime in the melt. These fundamental insights into the putative mechanisms of ion exchange and clustering were only accessible when our X-ray scattering experiments and AIMD simulations were put into context using the framework of a hybrid TS-Marcus theory, highlighting the importance of understanding the structure, dynamics, and energetics of the molten salt composition at the atomic level. Furthermore, our analysis shows that Cr^3+^ species possessing high charge density exhibit the slowest short-time dynamics among all constituent ions in the melt. Hence, during Cr dealloying/corrosion it is possible that concentration gradients will occur at the alloy-molten salt interface resulting in Cr^3+^-containing clusters, like those observed in this study, or even larger networks. These structures may be significantly different from those in the bulk and so will be the local chemical potentials conceivably affecting the dealloying rate. We expect that results presented here stemming from multiple experimental, theoretical and computational techniques will foster discussion on the issue of kinetics and aggregation in purely ionic media, with pertinence to novel molten salt reactors and emerging concentrated solar power technologies.

## Experimental section

### Sample preparation

The MgCl_2_ (anhydrous, >98%) was purchased from Sigma-Aldrich and further purified by fractional distillation described previously.^25,29^ The KCl (anhydrous, 99.999%) and CrCl_3_ (anhydrous, 99.9%) were both purchased from Sigma-Aldrich and used for experiments as received. Great care was taken so that the salts were maintained under a vacuum or inert atmosphere for the duration of sample preparation. Distilled MgCl_2_ and KCl were both added to a fused silica test tube inside an inert atmosphere glovebox (<0.5 ppm O_2_) at a 50 : 50 molar ratio. A vacuum valve compression fitting was then attached to the open end of the tube and the valve closed. This mixture was taken outside of the glovebox, evacuated to a pressure of 1.0 × 10^−3^ torr, and placed in a furnace at a temperature of 850 °C. Dynamic vacuum and temperature were maintained for 15 minutes to fuse the MgCl_2_–KCl mixture. The fused mixture was then cooled to room temperature under dynamic vacuum, sealed, and returned to the glovebox. Proper masses of both the MgCl_2_–KCl and CrCl_3_ salts were mixed to obtain the corresponding 5.0 mol% CrCl_3_ in the 50 : 50 MgCl_2_–KCl. This mixture was then fused together under dynamic vacuum following the same procedure as described for the MgCl_2_–KCl. The obtained salt compositions were then crushed to fine powder, added to separate thin-walled quartz capillaries (Charles Supper Co., 1.5 mm O.D., 0.010 mm wall thickness), evacuated to a pressure of 1.0 × 10^−3^ torr, and flame sealed. Additional details of the density measurements are provided in the ESI.†

### Synchrotron X-ray scattering experiments

Collection of total X-ray scattering data for PDF analysis took place at the 28-ID-1 beamline of the National Synchrotron Light Source II (NSLS-II), Brookhaven National Laboratory. A horizontally focusing side bounce monochromator was used to deliver X-rays with the energy of 74.4 keV (0.1667 Å) and a beam of cross-sectional area 0.25 × 0.25 mm^2^. An amorphous 2D silicon-based area detector (PerkinElmer XRD 1621, 200 × 200 micron pixels) was positioned about 20.8 cm from the sample to collect total scattering intensity data, which yielded a useable *Q* range up to 24 Å^−1^. A Ni powder standard was used to calibrate the sample to detector distance, beam center, and detector tilt and rotation. Measurements were performed in transmission mode using a customized furnace (ESI Fig. 2†) described in our previous study.^25^ The temperature was set using an Omega Engineering CN7800 series temperature controller. An Omega stainless-steel-sheathed K-type thermocouple was used to control a 24 volt DC TDK Lambda power supply. The furnace was temperature-calibrated using a K-type thermocouple inserted into a quartz tube (Charles Supper Co., 1.5 mm O.D., 0.010 mm wall thickness).

The X-ray scattering data were collected for the duration of 30 min for each sample and empty capillary. To minimize the overexcitation of pixels in the 2D detector, the duration of beam exposure was shorter for the crystalline samples than for the liquid salts at 1073 K. In addition, dark current scans were collected and subtracted from the raw X-ray patterns, allowing to remove residual intensity and prevent pixel overexcitation.^43^ After data collection, the GSAS-II program^44^ was used to radially integrate the 2D detector images to 1D diffraction patterns. The appropriate masks were applied to remove dead pixels and the beamstop from data integration. PDFgetX2 (ref. 45) was then used to subtract the background signal (empty quartz capillary and air scattering) and to perform additional corrections (sample self-absorption, multiple scattering, and inelastic Compton scattering) following standard procedures.^26,27^ The obtained data were subsequently normalized to the average electron density given by the weighted sum of the atomic X-ray form factors, yielding the total structure function, *S*(*Q*), which was then converted to PDF *via* Fourier transformation using a *Q* range of 0.5–18 Å^−1^ (see the ESI† for the corresponding equations).

### Optical spectroscopy measurements

All salt handling operations and optical spectroscopy measurements were performed inside an Ar atmosphere glovebox maintained at less than 0.1 ppm moisture and O_2_ concentrations. The absorption spectra were measured with the Cary 5000 spectrophotometer, equipped with deuterium and tungsten lamp covering the ultraviolet-visible-near infrared region over the wavelength range of 2300 to 200 nm. High temperature rated fiber optic cables procured from Fiberguide Inc. were used to couple the spectrophotometer (located external to the glovebox) to the custom designed furnace located outside the glovebox. To record the absorption spectra in transmission mode, molten salt samples were contained in a quartz cuvette (optical path length 1 cm, FireflySci) placed on a multi-cell rack designed with graphite; the furnace setup is described in our previous study.^46^ The wavelength scale of the spectrophotometer over the range of 880 to 280 nm was verified and calibrated using a NIST certified reference material consisting of an aqueous solution of didymium perchlorate that is permanently sealed by heat fusion in a high quality Far UV quartz cell. The salt samples were loaded in cleaned and dried quartz cuvettes. The total mass for each sample was approximately 4 g. A blank sample of base salt composition was used to calibrate the baseline of the instrument prior to measurement of the spectra at each temperature setup point.

### Raman spectroscopy experiments

High temperature Raman studies were carried out using a Horiba T64000 spectrometer. A diode laser of 532 nm was focused through a 50× objective of the microscope. The power of the laser was 100 mW. A Linkam heating stage TMS1100 was used for measuring Raman signal of molten salts at 1073 K. The stage was connected to a nitrogen flow (15 ml min^−1^) to keep the samples (50–80 mg) in alumina crucibles under an inert atmosphere during the measurements. The acquisition time was 10 s for each sample. The Raman shift was measured from 100 to 1200 cm^−1^, the range that should cover the expected Raman signal of MgCl_2_–KCl molten salt mixtures.^47,48^

### *Ab initio* molecular dynamics simulations

The initial structures for AIMD simulations were first pre-equilibrated by running force field-based simulations using the polarizable ion (PIM) model. The initial configuration for 50% MgCl_2_ + 50% KCl without CrCl_3_ was taken from our previous AIMD simulation that employed a different density functional.^25^ We used the average equilibrium density from the PIM model (*ρ* = 1.57 g cm^−3^) that is in close agreement with the experimental value (*ρ* = 1.56 g cm^−3^).^49^ Since there are no published PIM parameters for CrCl_3_, we constructed an initial system containing AlCl_3_, more specifically 6 Al^3+^, 60 Mg^2+^, 60 K^+^, and 198 Cl^−^ ions. We performed force field-based simulations of this system using the PIM model^25,50^ in the constant temperature–pressure ensemble, wherein we first heated the system at 1600 K at 1 bar for 100 ps and then equilibrated it at 1073 K keeping the same pressure for 500 ps. We extracted a configuration from this trajectory that has the experimental density of 1.65 g cm^−3^ (a total of 324 ions in a box with length of 22.3812 Å) and replaced Al with Cr before starting the AIMD simulations.

AIMD simulations were carried out using the Quickstep module of CP2K 6.1 (ref. 51) utilizing the PBE exchange–correlation functional^52–54^ in conjunction with Grimme's D3 dispersion correction.^55^ The MOLOPT-DZVP basis set^56^ in conjunction with Goedecker–Teter–Hutter (GTH) pseudopotentials^57^ was applied for all ions, together with a density cutoff of 600 Ry. All DFT calculations were performed with the Hubbard + *U* term (*U*_eff_ = *U* − *J* = 3.36 eV). Simulations were carried out using a time step of 0.5 fs and the temperature was set to 1073 K using a Nosé–Hoover thermostat^58,59^ with a velocity rescaling time constant of 1.0 ps. We obtained a 55 ps long AIMD trajectory and used the last 40 ps for the structural and dynamical analysis. Spin unrestricted calculations with several options for initial magnetizations were performed before the MD step to ensure that each Cr^3+^ has the expected spin state and the ground electronic state is identified. The orbital transformation method with a FULL_ALL preconditioner and a conjugate gradient minimizer was employed for achieving and accelerating the SCF convergence. Details of *S*(*Q*), *G*(*r*), and d*G*(*r*) calculations based on the AIMD trajectory are given in the ESI.†

### Reverse Monte Carlo modelling

The X-ray total scattering pattern was fitted through the RMC approach with the AIMD cell expanded to a 4 × 4 × 4 supercell, targeting an optimal match with experimental data in a metropolis manner. In our study, the RMCProfile package^60,61^ with such an algorithm implemented was employed to fit the X-ray total scattering data in both real and reciprocal space. A total of 20 736 ions in a cubic box with length of 89.5248 Å was used for the RMC fitting, with ∼493 moves per ion generated and ∼56 moves per ions accepted, overall. Apart from the experimental data, only the minimum distance for each ionic pair was used as constraint to allow the system to fully relax. To guarantee the generality of the obtained RMC results, the fit was performed using 50 different configurations and an average was taken over all RMC runs to obtain partial radial distribution functions, which were compared to our AIMD results.

## Author contributions

Project administration, A. S. I., C. J. M., V. S. B. Conceptualization and design of experiments, A. S. I. X-ray scattering experiments, A. S. I., M. A., S. K. G. and analysis, A. S. I. Raman measurements, A. S. I., D. S. M. Optical spectroscopy measurements, R. G. Sample preparation, P. H., D. S. M., S. M. M., S. D. AIMD simulations, V. S. B. Structure function, PDF, dPDF computation and analysis, C. J. M., S. S., W. V. K., F. W. RMC modelling, A. S. I., Y. Z. TS-Marcus theory model conceptualization, rate theory calculations and clustering analysis, S. R. Writing – original draft, A. S. I., C. J. M., S. R. All authors proofread, commented on, and approved the final version of the manuscript.

## Conflicts of interest

There are no conflicts to declare.

## Supplementary Material

SC-012-D1SC01224J-s001
