# Supplementary material for: X-ray scattering reveals ion clustering of dilute chromium species in molten chloride medium
Source: Chem Sci. 2021 May 19;12(23):8026–35. doi: 10.1039/d1sc01224j (PMC8208131; doi:10.1039/d1sc01224j)
Supplement: SC-012-D1SC01224J-s001 [file SC-012-D1SC01224J-s001.pdf]

Electronic Supplementary Material (ESI) for Chemical Science.
This journal is © The Royal Society of Chemistry 2021

X-ray Scattering Reveals Ion Clustering of Dilute Chromium Species in Molten Chloride Medium

Electronic Supporting Information

Santanu Roy,[a] Shobha Sharma,[b] Waruni V. Karunaratne,[b] Fei Wu,[b] Ruchi Gakhar,[c] Dmitry S. Maltsev,[d] Phillip Halstenberg,[a,d] Milinda Abeykoon,[e] Simerjeet K Gill,[f] Yuanpeng Zhang,[g] Shannon M. Mahurin,[a] Sheng Dai,[a,d] Vyacheslav S. Bryantsev,*[a] Claudio J. Margulis*[b] and Alexander S. Ivanov*[a]

[a]Chemical Sciences Division, Oak Ridge National Laboratory, Oak Ridge, TN 37831, USA
[b]Department of Chemistry, The University of Iowa, IA 52242, USA
[c]Pyrochemistry and Molten Salt Systems Department, Idaho National Laboratory, Idaho Falls, ID 83415, USA
[d]Department of Chemistry, University of Tennessee, Knoxville, TN 37996, USA
[e]National Synchrotron Light Source II (NSLS-II), Brookhaven National Lab, USA
[f]Chemistry Division, Brookhaven National Lab, Upton, New York 11973, USA
[g]Neutron Scattering Division, Oak Ridge National Laboratory, Oak Ridge, TN 37831, USA

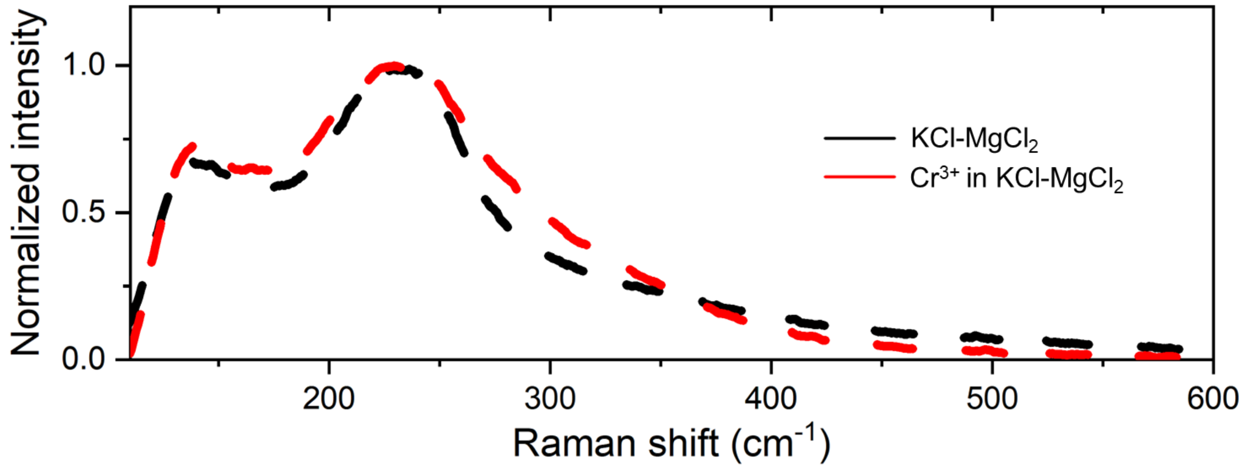

Figure ESI-1 Raman spectra for the KCl-$MgCl_2$ (50:50 mol%) molten salt (black dashed line) and $CrCl_3$ (5 mol%) in the KCl-$MgCl_2$ (red dashed line) at 1073 K. Note that the addition of dilute Cr^{3+} ions slightly modifies the KCl-$MgCl_2$ spectrum in the ~260-350 cm^{-1} region, however, it is impossible to clearly assign frequencies related to Cr^{3+} species in the molten salt, revealing the limitations of this form of spectroscopy for investigation of dilute metal ion species. The most intense band at ~232 cm^{-1} corresponds to the Mg–Cl symmetric stretching vibrations based on the previous reports.[1,2]

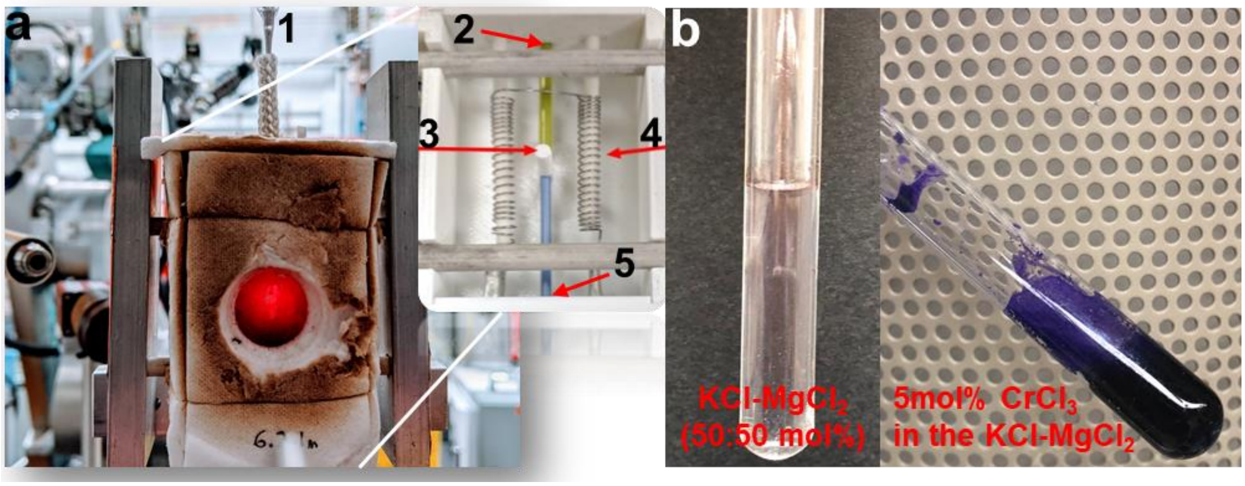

Figure ESI-2 Furnace and molten salt samples used for X-ray scattering studies at NSLS-II. a) Experimental setup at the PDF beamline, NSLS-II (the inset shows inside features of the furnace for clarity): 1, molten salt sample in quartz capillary; 2, sample port; 3, X-ray entrance port; 4, heating wire; 5, thermocouple port. b) Molten KCl-$MgCl_2$ (50:50 mol%) (left) and $CrCl_3$ (5 mol%) in the KCl-$MgCl_2$ (right) salts at ~1073 K.

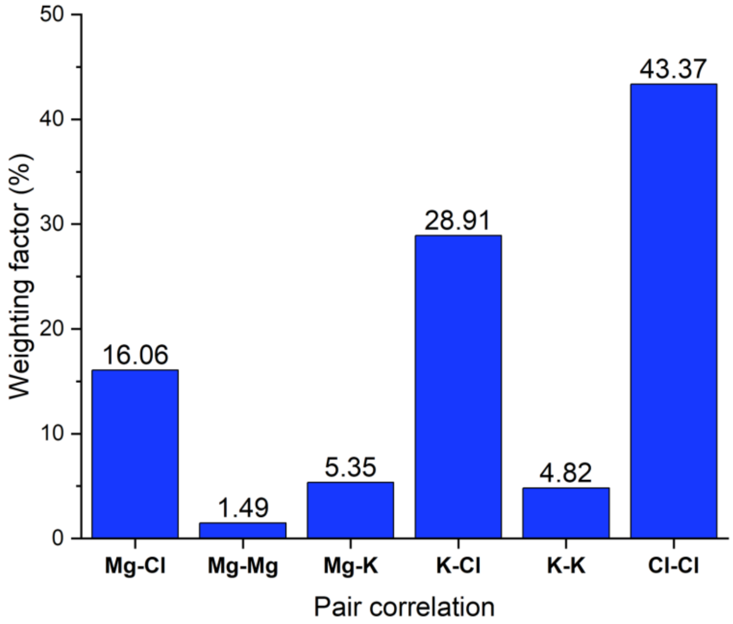

Figure ESI-3 The relative X-ray weighting factors ($w_{ij}(Q) = \frac{(2-\delta_{ij})x_i x_j f_i(Q) f_j(Q)}{[\sum_i x_i f_i(Q)]^2}$, where δ_{ij} is one for i=j and zero for i≠j) calculated at Q=0 for the ionic pair correlations in the KCl-$MgCl_2$ system. As can be seen, most of the scattering comes from the pairs containing chlorides and thus the X-ray diffraction patterns in Fig. 1 (main text) are primarily dominated by Cl-Cl, K-Cl, and Mg-Cl correlations.

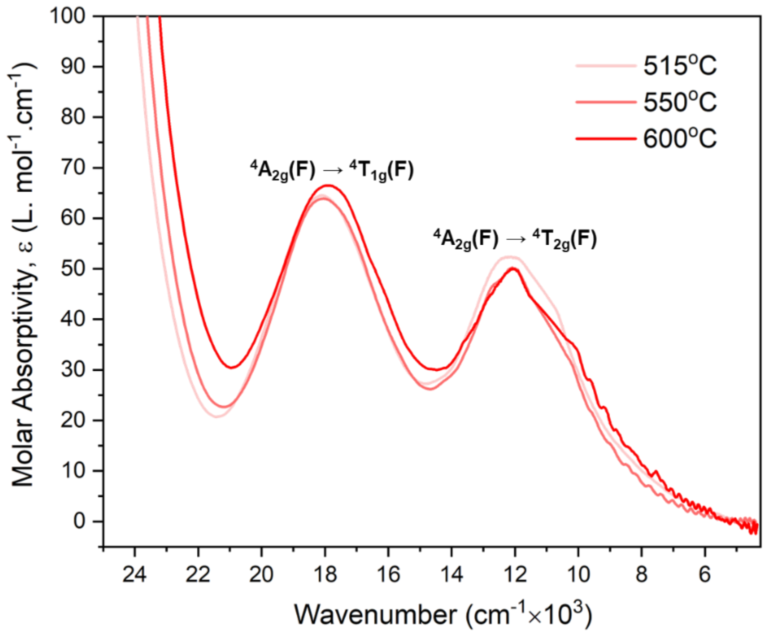

Figure ESI-4 Optical absorption spectra of Cr^{3+} (5 mol%) in KCl-$MgCl_2$ (50:50 mol%), as a function of temperature. It is a well-defined spectrum with two bands in the visible region, assigned as follows: to $^4A_2 \rightarrow {}^4T_2$ (^4F) for peak at 12,000 cm^{-1}, and to $^4A_2 \rightarrow {}^4T_1$($^4F$) for peak at 18,000 cm^{-1}, and a charge transfer band corresponding to $^4A_2 \rightarrow {}^4T_1$($^4P$) transition. There are no prominent features in the near-IR range. Based on the spectra, the symmetry of Cr^{3+}can be

attributed to the octahedral $CrCl_6^{3-}$ ions. The spectral features and hence the geometry of the complex remain consistent over the temperature range investigated. The results obtained are in agreement with the Cr^{3+}spectrum reported by Harrington and Sundheim,[3] and by Gruen and McBeth,[4] in the LiCl-KCl eutectic melt.

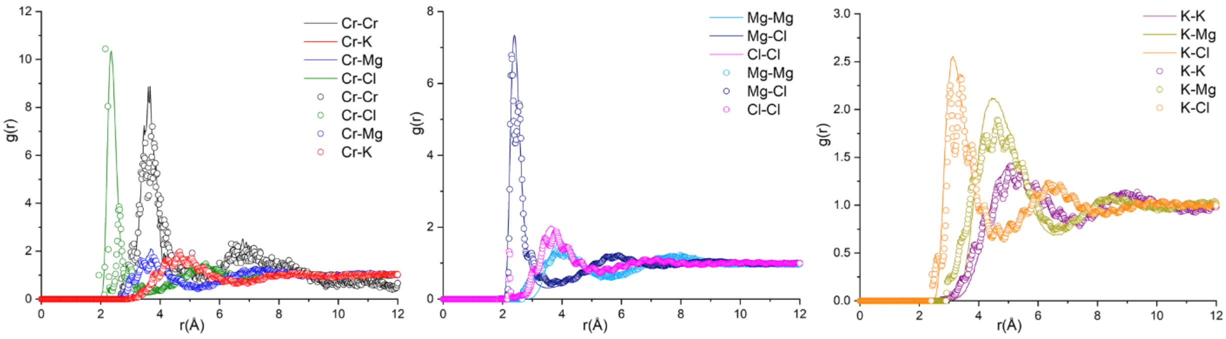

Figure ESI-5 Comparison of radial distribution functions, g(r), obtained from RMC modeling (circles) and AIMD simulations (solid lines) for the $CrCl_3$-KCl-$MgCl_2$ system.

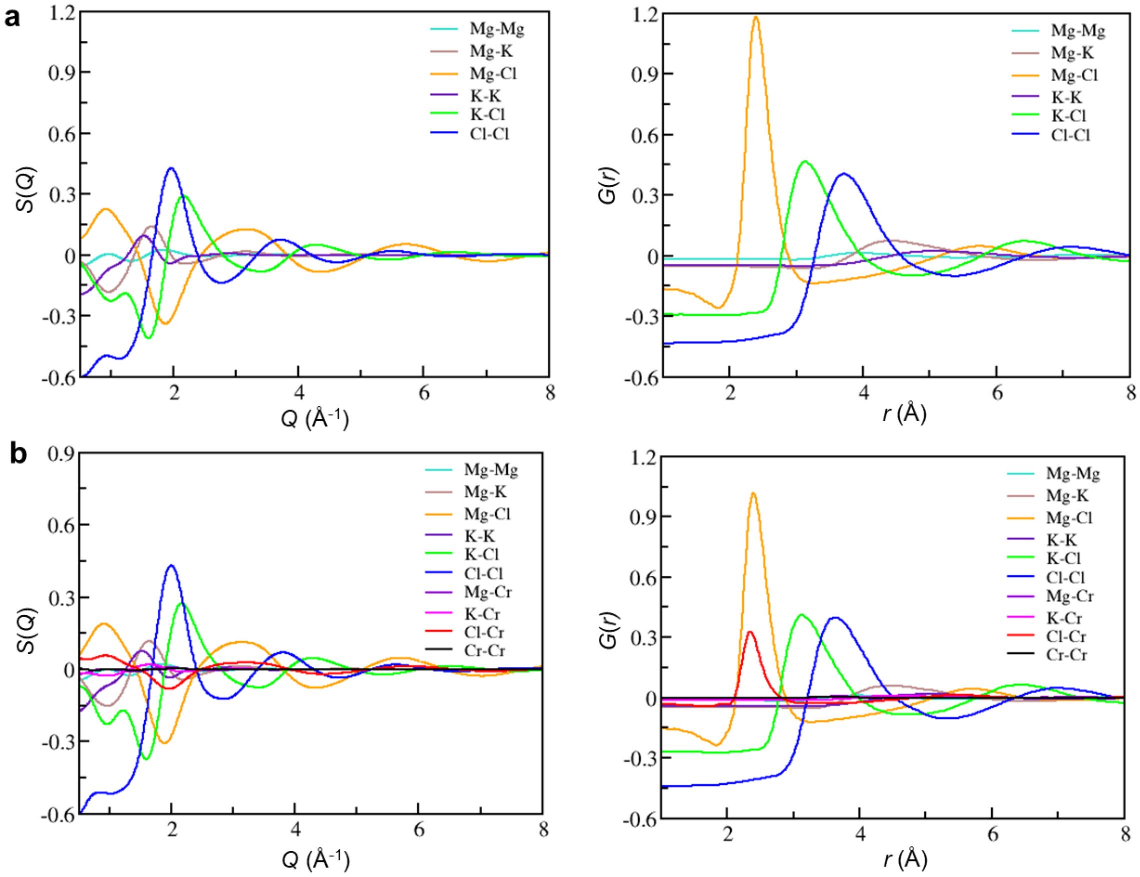

Figure ESI-6 Partial structure functions, $S(Q)$s, and pair distribution functions, $G(r)$s, obtained from the AIMD simulations for a) the KCl-$MgCl_2$ and b) the $CrCl_3$-KCl-$MgCl_2$ molten salts at 1073 K.

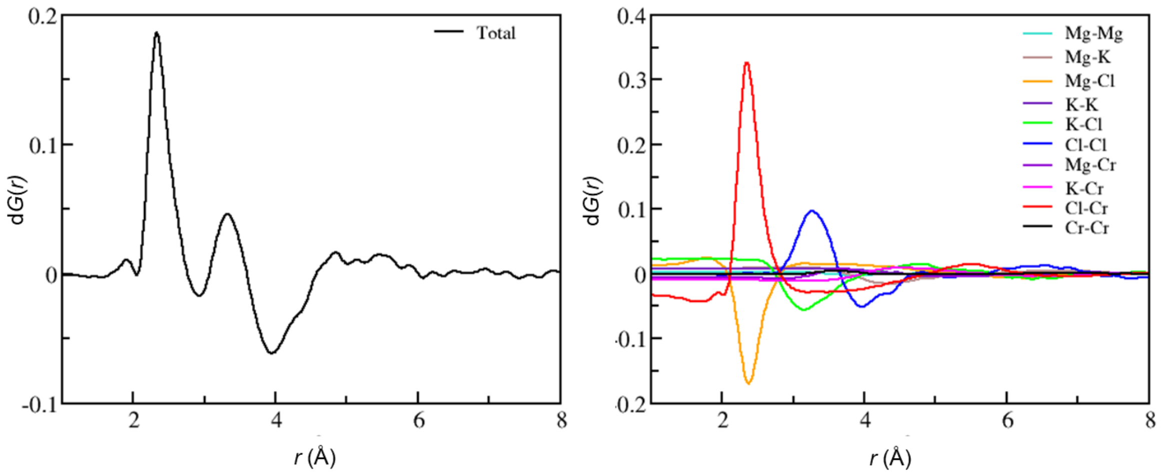

Figure ESI-7 The differential pair distribution function, d$G(r)$, and its partial subcomponents obtained from the AIMD simulations.

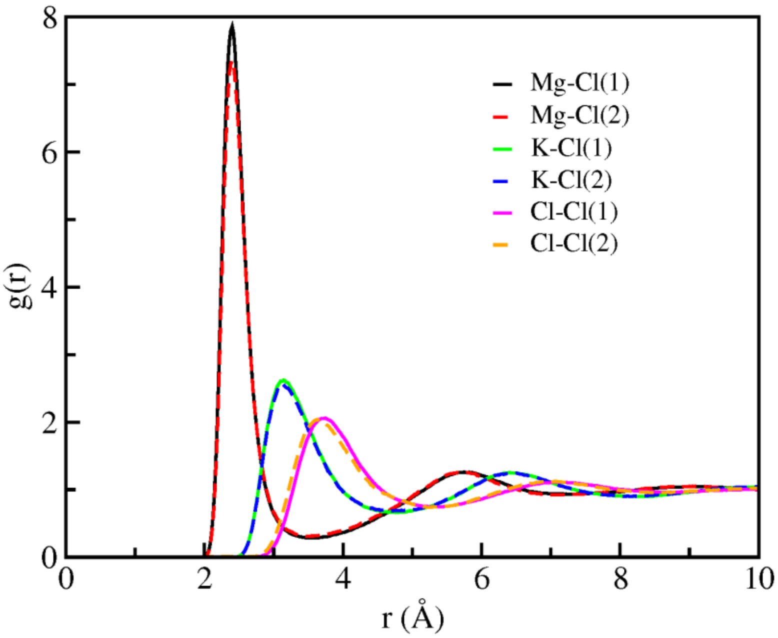

Figure ESI-8 Radial distribution functions from the AIMD trajectories for the molten salt systems without (1) and with (2) $CrCl_3$. Note a slight shift of the Cl-Cl g(r) toward shorter distances because of the presence of Cr^{3+} in the melt.

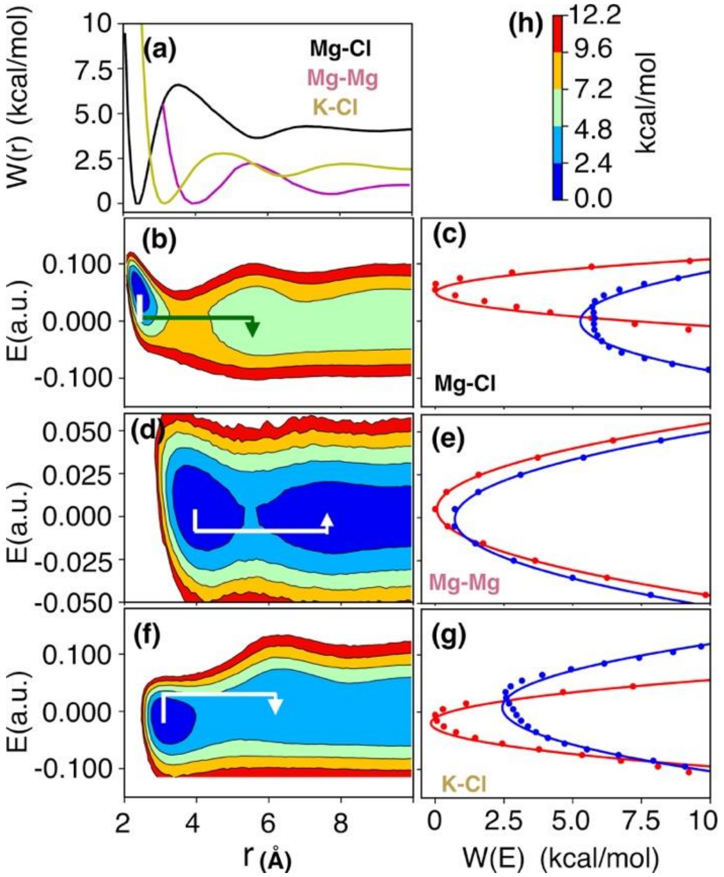

Figure ESI-9 1D (a) and 2D (b,d,f) free energies (involving Mg-Cl, Mg-Mg, K-Cl distance and electric field (in atomic unit) experienced by Cl, Mg, and Cl, respectively) and corresponding Marcus parabolas (solids are parabolic fits and dots are actual data) in electric field space (c,e,g) describing chloride exchange around Mg and dissociation of Mg-Mg dimers and chloride exchange around K. Reorganization of ionic media that causes electric field rearrangement drives exchange and dissociation events. The color bar (h) represents the contour levels in the 2D-free energy surfaces.

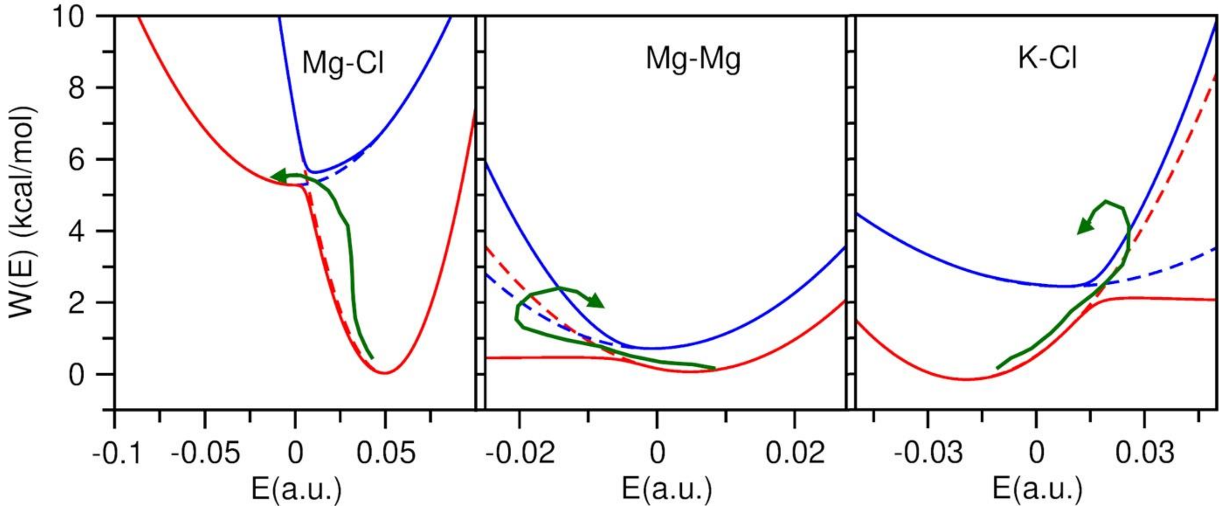

Figure ESI-10 Lower and higher adiabatic energy surfaces (solids) obtained from coupled Marcus diabats (dots) in electric field space and the reactant-to-product transition path highlighted by a green arrow. While the chloride exchange around Mg follows an adiabatic path (transition dynamics confined on the lower adiabatic surface), the dissociation of Mg-Mg dimmer and chloride exchange around K are described by a nonadiabatic path (transition dynamics requires hopping on the higher energy surface).

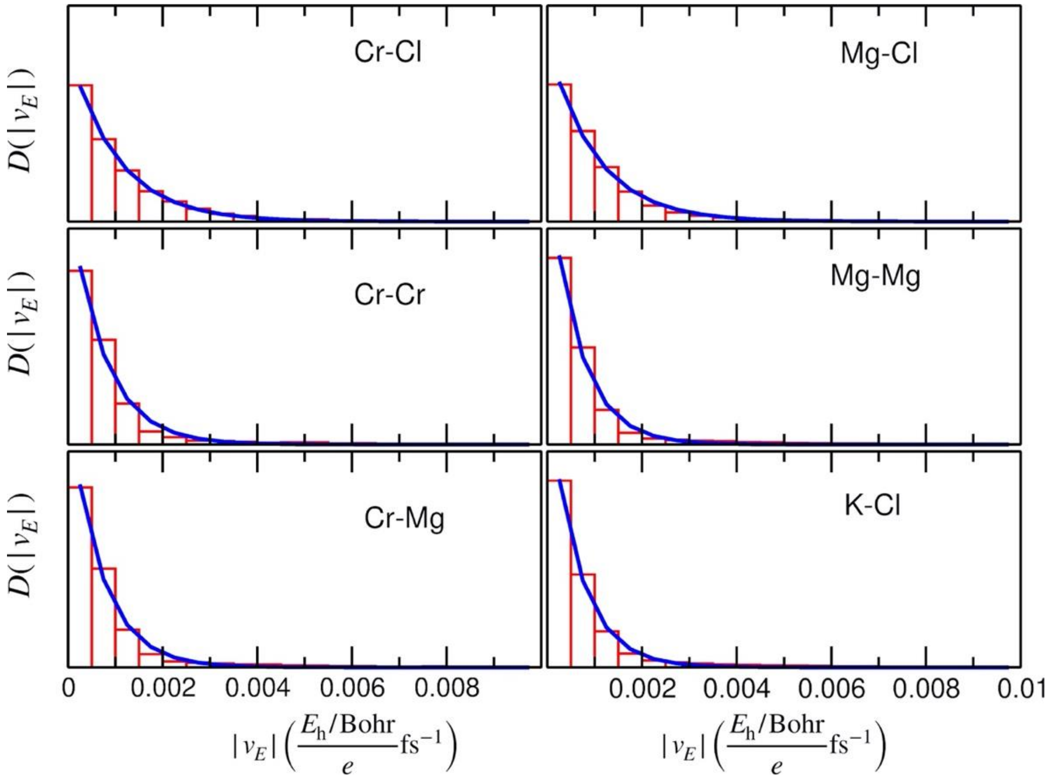

Figure ESI-11 Distributions of velocities at which electric field trajectories cross the transition state at $E=E^{\dagger}$ for different scenarios of chloride exchange around cations and dissociation of cation-cation chloride-shared dimers.

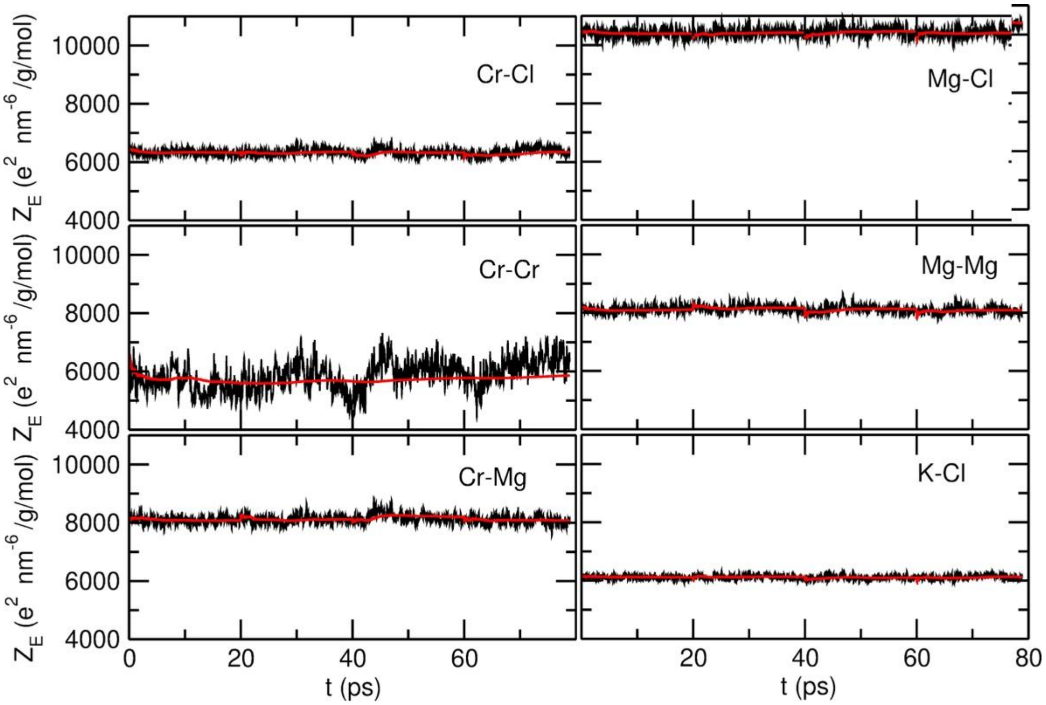

Figure ESI-12 Inverse of a mass-like quantity associated with the motion along electric field, which is conserved through the simulation (red indicates running average).

Table ESI-1 Parameters of the hybrid TS-Marcus theory for chloride exchange around Cr^{3+} (Cr-Cl), Mg^{2+} (Mg-Cl), and K^+ (K-Cl) and dissociation of dimeric Cr-Cr, Cr-Mg, and Mg-Mg configurations.

	Cr-Cl	Cr-Cr	Cr-Mg	Mg-Cl	Mg-Mg	K-Cl
Marcus parabolas						
W_R	-0.1628	-0.04398	0.13201	0.027607	0.065153	-0.15785
W_P	5.8357	2.89793	1.06426	5.27748	0.714734	2.44279
(kcal/mol)						
E_R	0.09465	0.03039	0.01991	0.04959	0.004876	-0.019397
E_P	0.01467	0.01058	0.00521	-0.00031	-0.00081	0.007957
$\left(\frac{E_h/\text{Bohr}}{e}\right)$						
K_R	5374.0	7156.64	6443.04	5818.95	7865.78	3551.44
K_P	1016.4	4877.38	6857.34	1255.14	7154.03	1220.11
$\left[\text{kcal mol}^{-1}/\left(\frac{E_h/\text{Bohr}}{e}\right)^2\right]$						

Barriers						
$W(E^{\dagger})$	4.548	2.259	0.943	4.891	0.465	2.058
$\Delta W_r^{E^{\dagger}}$	5.900	4.220	2.600	4.462	1.900	1.680
$\left[W(E^{\dagger}) + \Delta W_r^{E^{\dagger}}\right]$ (kcal/mol)	10.448	6.479	3.543	9.353	2.365	3.738
$e^{-\beta\left[W(E^{\dagger})+\Delta W_r^{E^{\dagger}}\right]}$	0.00745	0.047908	0.18987	0.01244	0.329825	0.173227
Mass-weighted reactant volume						
Z_E (e^2 nm^{-6}/g mol^{-1})	6334.423 ± 117.000	5859.540 ± 500.000	8116.252 ± 151.000	10418.30 ± 152.500	8116.15 ± 127.70	6114.29 ± 75.00
$\frac{1}{\sqrt{Z_E}} V_{\mathrm{R}}^{E}$ (nm $g^{1/2}$ $mol^{-1/2}$)	0.312 ± 0.003	0.216 ± 0.009	0.194 ± 0.002	0.198 ± 0.001	0.215 ± 0.002	0.105 ± 0.001
Barrier-recrossing						
κ_{LZ}	0.999	0.116	0.210	0.552	0.009	0.353
C (kcal/mol)	2.421	0.900	0.143	0.551	0.665	0.533
$\lvert\bar{v}_E\rvert$ $\left(\frac{E_h/\mathrm{Bohr}}{e}\, fs^{-1}\right)$	0.001029	0.000733	0.000712	0.001016	0.000645	0.000644
$\lvert S_2 - S_1\rvert$ (kcal mol^{-1}/ $\frac{E_{\mathrm{h}}/\mathrm{Bohr}}{e}$)	295.4975	164.6591	93.69809	257.0060	52.36781	123.9671
Timescale: τ (ps)						
No barrier-recrossing	35.2±0.3	3.8±0.2	0.9±0.01	13.2±0.1	0.5±0.004	0.5±0.003
With barrier-recrossing	35.2±0.3	32.6±1.5	4.1±0.04	24.1±0.2	60.6±0.50	1.44±0.01

Density measurements. Given the bright purple color of the KCl-$MgCl_2$ molten salt after addition of 5 mol% $CrCl_3$ (Fig. ESI-2b), the density of the resulting $CrCl_3$-KCl-$MgCl_2$ mixture can be accurately determined by measuring the volume of the melt at 800^0C. A similar technique

to measure the density of molten NaCl-$CrCl_3$ salt was applied by Li et al.[5] To determine the volume, the salt mixture was melted under an inert atmosphere in a calibrated quartz tube. The molten salt visibly colored the quartz and the colored part of the tube was used to deduce the height, from which the volume can be calculated for the molten salt sample of known mass. The measurements were repeated three times with different amounts of the $CrCl_3$-KCl-$MgCl_2$ salts, giving the density of 1.65 g/cm^3 at 800^{0}C.

X-ray structure function and pair distribution function. The X-ray structure function, $S(Q)$, is defined as the following:

$$S(Q) = \frac{I_{coh}(Q) - \sum_i x_i f_i^2(Q)}{[\sum_i x_i f_i(Q)]^2} \quad (1)$$

where I_{coh} is coherent scattering intensity, x_i and $f_i(Q)$ are the molar fraction and Q-dependent X-ray ionic form factor of species i, respectively, and Q denotes the magnitude of the scattering vector ($Q = 4\pi \sin(\theta)/\lambda$), where 2θ is the scattering angle, and λ is the incident X-ray wavelength. Notice that as defined here, $S(Q)$ goes to zero at large Q. In other words, our $S(Q)$ is $F^X(Q)$ as defined in the review article by Keen;[6] we highlight this to avoid confusion with different terminologies and definitions commonly used in total scattering.

Computationally we define the X-ray weighted total scattering structure function, $S(Q)$, as

$$S(Q) = \frac{\rho_0 \sum_i \sum_{j \geq i} x_i\, x_j f_i(Q) f_j(Q) \int_0^\infty 4\pi r^2 \left(g_{ij}(r) - 1\right) \frac{sin(Qr)}{Qr} dr}{[\sum_i x_i f_i(Q)]^2} \quad (2)$$

Where ρ_0 is the ionic number density of the system, x_i and x_j are molar fractions of ionic species i and j, $f_i(Q)$ and $f_j(Q)$ are X-ray atomic form factors for species i and j and $g_{ij}(r)$ is the radial pair distribution function for species i and j.

Real space pair distribution functions (PDF), $G(r)$ and $D(r)$, are obtained from $S(Q)$ via the expressions

$$G(r) = \frac{1}{(2\pi)^3 \rho_0} \int_0^\infty 4\, \pi q^2 S(Q) \frac{\sin(Qr)}{Qr} dQ \quad (3)$$

$$D(r) = 4\pi\rho_0 r G(r) = \frac{2}{\pi} \int_0^\infty Q S(Q) \sin(Qr)\, dQ \quad (4)$$

One can also define the partial subcomponents of $G(r)$ and $D(r)$ via the Fourier transformation of the partial subcomponents of $S(Q)$.

$$S_{ij}(Q) = 2 \times \frac{\rho_0 x_i x_j f_i(Q) f_j(Q) \int_0^\infty 4\pi r^2 \left(g_{ij}(r) - 1\right) \frac{\sin(Qr)}{Qr} dr}{[\sum_i x_i f_i(Q)]^2}$$

$$S_{ii}(Q) = \frac{\rho_0 x_i x_i f_i(Q) f_i(Q) \int_0^\infty 4\pi r^2 (g_{ii}(r) - 1) \frac{sin\,(Qr)}{Qr} dr}{[\sum_i x_i f_i(Q)]^2} \quad (5)$$

and

$$G_{ij}(r) = \frac{1}{(2\pi)^3 \rho_0} \int_0^\infty 4\pi Q^2 S_{ij}(Q) \frac{\sin(Qr)}{Qr} dQ$$

$$D_{ij}(r) = 4\pi\rho_0 r G_{ij}(r) \quad (6)$$

These provide information as to which pair interactions contribute to the PDF at specific distances. The differential PDF, $\mathrm{d}G(r)$, is accordingly defined as in Eq. 3, but with $S(Q)$ replaced by the difference between two $S(Q)$s for the $CrCl_3$-loaded KCl-$MgCl_2$ and the pristine KCl-$MgCl_2$ molten salt mixture. Note that a direct subtraction of $G(r)$ for the KCl-$MgCl_2$ mixture from $G(r)$ for the $CrCl_3$-KCl-$MgCl_2$ leads to the identical $\mathrm{d}G(r)$ as follows from the above definitions.

Coordination number. If r_i is the distance between the ith Cl^- ion out of a total number of Cl^- ions (N_{Cl}) and a cation, and $r^\dagger$ is the location of the boundary of the first chloride solvation shell determined from the first minimum of the cation–Cl^- radial distribution function (RDF), the coordination number of the cation is defined in terms of a smooth function, f_i, ($0 < f_i < 1$):

$$CN = \sum_{i=1}^{N_{\mathrm{Cl}}} \frac{1 - \left(\frac{r_i}{r^\dagger}\right)^{12}}{1 - \left(\frac{r_i}{r^\dagger}\right)^{24}} = \sum_{i=1}^{N_{\mathrm{Cl}}} f_i \quad (7)$$

Eq. 7 allows smooth transitions of Cl^- across the boundary of the first chloride solvation shell.

Number of shared Cl^-. f_i in Eq. 1 represents the contribution of the ith Cl^- to the coordination structure of a cation. Thus, if f_{ij} and f_{ik} are the contributions of the ith Cl^- respectively to the coordination structures of the jth and k^{th} cations, the number of Cl^- shared between these two cations is given by:

$$n_{\mathrm{Shared}} = \sum_{i=1}^{N_{\mathrm{Cl}}} f_{ij} f_{ik} \quad (8)$$

Notice that, n_{Shared} is maximum for Cl^- ions that are located between these two cations, contributing the most to their shared overlapping coordination shells. Eq. 8 can also be used to determine the number of cations coordinating with two different cations through chloride ions, but in this case, f is determined using the cation–cation RDF and corresponding $r^\dagger$.

Free energy calculations. The 1D-free energy profile for a reaction coordinate is calculated using its probability distribution function (Ω (x)) computed from the AIMD trajectory: $W(x) = -k_B T ln[\Omega\,(x)]$, where x is the reaction coordinate such as coordination number (CN) or distance (r). The 2D-free energy surfaces were computed using the joint probability distribution function

(Ω (*r*,*y*)): $W(r,y) = -k_B T ln[\Omega(r,y)]$, where y is a coordination number (*CN*), or number of shared chloride ion between two cations, or number of cations simultaneously coordinating with two different cations via chloride ions (n_{Shared}), or electric field (*E*). Note that the averaging for the free energies was done considering all possible ion pairs and the production length of the AIMD trajectories.

TS-Marcus approach. We describe rate processes in a Coulombic system of molten salts in terms of the reaction coordinate, *E*, which is the electric field exerted by the solvent ions on the solute ion projected along a specific direction ($\hat{u}$), and the ionic solvent bath coordinate, *B*. Following the work of Darve and Pohorille,[7] the Hamiltonian for this system can be determined through a coordinate transformation from a set of conventional Cartesian coordinates *X* of 3*N* components (*N* is the total number of ions) to the set of (*E*, *B*), where *B* has 3*N*−1 components. The associated conjugate momenta in this new set are p_E and P_B with 3*N*−1 components—these are also transformed from the Cartesian momenta P_X with 3*N* components. Thus, the Hamiltonian can be expressed as: $H = \frac{1}{2} Z_E p_E^2 + \frac{1}{2} P_B^T Z_B P_B + V(E,B)$ (9)

The first two terms in the above equation represent respectively the kinetic energy associated with the motion of the electric field and bath coordinates (notice that the cross-terms between p_E and P_B have been ignored for simplicity) and the last one is the potential energy. $1/Z_E$ is a mass-like quantity moving with the momentum p_E in the electric filed space and can be obtained as: $Z_E = \sum_{i=1}^{3N} \frac{1}{m_i}\left(\frac{\partial E}{\partial x_i}\right)^2$. Here, x_i is a component of the Cartesian coordinate *X* and m_i is the associated mass. If the ith solvent ion with charge Q_i (considering formal charges +3, +2, +1, and -1 respectively for Cr^{3+}, Mg^{2+}, K^+, and Cl^-) is located at a distance r_i, the electric field on a solute ion exerted by all the solvent ions projected along $\hat{u}$ is obtained as $E = \hat{u}.\sum_{j=1}^{N-1} \frac{Q_i}{{r_i}^2}\hat{r_i}$. Thus, Z_E takes the form of:

$$Z_E = \sum_{i=1}^{N-1} \frac{(f_i')^2}{\mu_i} + \frac{2}{M} \sum_{i=1}^{N-2} \sum_{j=i+1}^{N-1} f_i' f_j' \left(\hat{r_i}.\hat{r_j}\right) \qquad (10)$$

where $f_i' = \frac{dE}{dr_i}$ and $f_j' = \frac{dE}{dr_j}$, and M and μ_i are the mass of the solute ion and the solute ion-i^{th} solvent ion reduced mass, respectively. $\hat{r_i}$ and $\hat{r_j}$ are the unit vectors pointing respectively from the ith and j^{th} solvent ions to the solute ion. Now, the rate of transition between an initial (E_i) and final (E_f) electric field states via the transition state, $E=E^\dagger$, can be obtained as:[8]

$$k_E = \frac{\int dB\, dp_B dE dp_E \delta(E - E^\dagger) Z_E p_E \Theta(Z_E p_E) e^{-\beta H}}{\int dB\, dp_B dE dp_E e^{-\beta H}}$$

$$= \sqrt{\frac{Z_E}{2\pi\beta}} \frac{e^{-\beta W(E^\dagger)}}{\int_{E_i}^{E^\dagger} dE\, e^{-\beta W(E)}}. \qquad (11)$$

Here, Θ is the Heaviside step function ensuring a positive flux ($Z_E p_E \geq 0$) through the transition state. $\beta = 1/k_B T$ is the inverse thermal energy at temperature T and k_B is the Boltzmann constant.

$W(E)$ is the potential of mean force (PMF) in the electric field space. Note that, TST assumes that after arrival at the transition state (barrier-top) from the reactant minimum on the PMF, a trajectory immediately moves to the product minimum. However, numerous studies on solvation and ion/charge transport indicated that solvent bath-induced barrier-recrossing is inevitable and must be accounted for to determine correct transition rates. The methods of reactive flux by Chandler,[9] Kramer's theory,[10] Grote-Hynes theory,[11] and the semiclassical Landau-Zener[12] approach are typically employed to examine such non-equilibrium solvent effects, wherein transmission coefficient is determined as a measure of the fraction of the flux of the trajectories through the transition state that finally arrives at the product minimum. The product of the TST rate and the transmission coefficient provides the correct rate of transition.

Recently, Marcus theory of electron transfer has been extended by Roy et al. to investigate ion pairing, solvent exchange, and ion exchange processes in condensed phase systems.[13-17] Following this theory, we can express the reactant (R) and product (P) free energy states using parabolic functions of electric fields, $W_{\mathrm{R}}(E)$ and $W_{\mathrm{P}}(E)$, respectively:

$$W_{\mathrm{R}}(E) = W_{\mathrm{R}} + \frac{1}{2}K_{\mathrm{R}}(E - E_{\mathrm{R}})^2$$

$$W_{\mathrm{P}}(E) = W_P + \frac{1}{2}K_{\mathrm{P}}(E - E_{\mathrm{P}})^2 \quad (12)$$

Here, W_{R} and W_{P} are the minima of the reactant product parabolas located at $E = E_{\mathrm{R}}$ and $E = E_{\mathrm{P}}$, and K_{R} and K_{P} are corresponding curvatures. These parabolas are diabatic states and the reactant-to-product transition occurs through their crossing locations. These diabats can be extracted as slices from a 2D-free energy surface (W(r,E)) spanned by interionic distance (r) and electric field. The first slice is for the equilibrium close-contact distance in the reactant state ($r = r_{\mathrm{R}}$) and the second one is for the equilibrium solvent-separated distance in the product state ($r = r_{\mathrm{P}}$). These slices, which cross at $E = E^{\dagger}$, are modeled with parabolic functions as presented in Eq. 12. An exact Marcus theory suggests that solvent reorganization in the form of electric field change drives the equilibrium reactant state, e.g., the state of close-contact ion pair, to an activated transition state (the crossing point of the two parabolas; $E = E^{\dagger}$) where the barrier along the distance ($\Delta W_r^{E^{\dagger}}$) are expected to vanish or reduce significantly, resulting in rapid dissociation of the ion pair, i.e., rapid transition to the equilibrium product state. However, in practice, slower transition rates are anticipated due to a large value of $\Delta W_r^{E^{\dagger}}$ and recrossing events of the crossing point. Then depending on specific cases, in addition to accounting for this additional barrier, the recrossing events are treated with either the adiabatic or nonadiabatic prescription of Marcus theory.

When the reactant and product parabolas start to couple (with the coupling strength C) as they start to cross, they are modified by each other resulting in a lower (W_{-}) and a higher (W_{+}) adiabatic free energy surfaces: $W_{\pm} = \frac{W_{\mathrm{R}}(E)+W_{\mathrm{P}}(E)}{2} \pm \frac{1}{2}\sqrt{4[C(E)]^2 + [W_{\mathrm{R}}(E) - W_{\mathrm{P}}(E)]^2}$. The coupling constant can be determined as: $C = \frac{K_{\mathrm{R}}+K_{\mathrm{P}}}{2\sqrt{K_{\mathrm{R}}K_{\mathrm{P}}}}\sqrt{[W_{\mathrm{R}}(E) - W_{\mathrm{R}}][W_{\mathrm{P}}(E) - W_{\mathrm{P}}]}$, where the prefactor disappears for $K_{\mathrm{R}} = K_{\mathrm{P}}$, i.e., when both the parabolas have equal curvatures.[13-17]

When the equilibrium locations of the reactant and product parabolas (diabatic states) are well-separated and cross at the "normal" region (opposite side), a strong coupling strength at the crossing point can create a large gap between the lower and higher adiabatic states. Then the reactant-to-product transition dynamics can be simply confined on the lower adiabatic surface. On the other hand, when the reactant and product parabolas cross at the "inverted" region (same side) with a weak coupling strength, a nonadiabatic jump from the lower to the higher surface is needed to describe the reactant-to-product transition. The crossing location affects the recrossing events, which is determined in terms of the transmission coefficient (i.e., the fraction of the flux of the trajectories that actually traverse the crossing point and land up at the product minimum). Herein, we determine the transmission coefficient using the semiclassical approach of Landau and Zener (κ_{LZ}).[12] κ_{LZ} is governed by the probability (P) of the reactive transitions through the crossing point: $\kappa_{\mathrm{LZ}} = 2P/(P+1)$ when the parabolas cross at the "normal" region and $\kappa_{\mathrm{LZ}} = 2P(1-P)$ when the parabolas cross at the "inverted" region. P depends on the coupling strength between the parabolas and the mean traversal velocity of the electric field ($\bar{v}_E$) at the crossing point, $E^{\dagger}$: $P = 1 - \exp\left[-\frac{2\pi[C(E^{\dagger})]^2}{\hbar|\bar{v}_E||S_2-S_1|}\right]$, where $\hbar = h/2\pi$ (h is the Planck constant) and $S_{1,2} = \frac{dW(E)}{dE}\Big|_{E=E^{\dagger}}$ are the slopes of the parabolas at the crossing point. The trajectories traversing the crossing point exhibit an exponential velocity distribution (Fig. ESI-11) in the form of: $D(|\mathrm{v}_E|) = \mathrm{D}_0 \exp[-|v_E|/|\bar{v}_E|]$, providing the mean velocity required to determine P.

To determine the rate of the reactant-to-product transition within a hybrid TS-Marcus framework, we can utilize the TST expression given in Eq. 11, the adiabatic energy surfaces obtained from coupled Marcus parabolas, and the transmission coefficient κ_{LZ}, providing a new rate expression:

$$k_E = \kappa_{\mathrm{LZ}} \sqrt{\frac{Z_E}{2\pi\beta}} \frac{e^{-\beta\left[W(E^{\dagger})+\Delta W_r^{E^{\dagger}}\right]}}{\int_{E_i}^{E^{\dagger}} dE\, e^{-\beta W(E)}}$$

$$= \kappa_{\mathrm{LZ}} \sqrt{\frac{1}{2\pi\beta}} \frac{e^{-\beta\left[W(E^{\dagger})+\Delta W_r^{E^{\dagger}}\right]}}{\frac{1}{\sqrt{Z_E}}\int_{E_i}^{E^{\dagger}} dE\, e^{-\beta W(E)}}$$

$$= \kappa_{\mathrm{LZ}} \sqrt{\frac{1}{2\pi\beta}} \frac{e^{-\beta\left[W(E^{\dagger})+\Delta W_r^{E^{\dagger}}\right]}}{\frac{1}{\sqrt{Z_E}} V_{\mathrm{R}}^{E}}, \qquad (13)$$

where, $W(E)=W_{-}(E)$, is the lower adiabatic free energy surface. Notice that $\frac{1}{\sqrt{Z_E}}\int_{E_i}^{E^{\dagger}} dE\, e^{-\beta W(E)} = \frac{1}{\sqrt{Z_E}} V_{\mathrm{R}}^{E}$ is a mass-weighted configuration space region in electric field—we call it "mass-weighted reactant volume", because the integral sums over the Boltzmann-weighted volume elements of electric field (dE) in the reactant region. Z_E computed for our systems are given in Fig. ESI-12. We accounted for the additional barrier, $\Delta W_r^{E^{\dagger}}$, that may exist

along the distance coordinate at $E = E^{\dagger}$. This barrier is determined by extracting a slice along $E = E^{\dagger}$ from the 2D-free energy surfaces and computing the barrier height from the reactant minimum of the slice.

References:

1. C. H. Huang and M. H. Brooker, *Chem. Phys. Lett.*, 1976, **43**, 180-182.
2. S. Dai, G. M. Begun, J. P. Young and G. Mamantov, *J. Ram. Spect.*, 1995, **26**, 929-932.
3. B. R. Sundheim and G. Harrington, *J. Chem. Phys.*, 1959, **31**, 700-701.
4. D. M. Gruen and R. L. McBeth, *Pure Appl. Chem.*, 1963, **6**, 23-48.
5. Q. J. Li, D. Sprouster, G. Zheng, J. Neuefeind, A. Braatz, J. McFarlane, D. Olds, S. Lam, J. Li and B. Khaykovich, 2020, DOI: 10.26434/chemrxiv.12971966.v1.
6. D. A. Keen, *J. Appl. Cryst.*, 2001, **34**, 172-177.
7. E. Darve, and A. Pohorille, *J. Chem. Phys.*, 2001, **115**, 9169-9183.
8. G. Schenter, B. Garrett and D. Truhlar, *J. Chem. Phys.* 2003, **119**, 5828-5833; S. Roy, M. D. Baer, C. J. Mundy and G. K. Schenter, *J. Phys. Chem. C*, 2016, **120**, 7597-7605
9. B. Berne, *Theoretical Chemistry Accounts*, 2000, **103**, 335-336.
10. S. Tucker, *Theoretical Chemistry Accounts*, 2000, **103**, 209-211.
11. R. Grote and J. Hynes, *Journal of Chemical Physics*, 1980, **73**, 2715-2732.
12. M. Newton and N. Sutin, *Annual Review of Physical Chemistry*, 1984, **35**, 437-480.
13. S. Roy, M. D. Baer, C. J. Mundy and G. K. Schenter, *J. Chem. Theory Comput.*, 2017, **13**, 3470-3477.
14. S. Roy, M. Galib, G. K. Schenter and C. J. Mundy, *Chem. Phys. Lett.*, 2017, **692**, 407-415.
15. S. Roy, F. Wu, H. Wang, A. S. Ivanov, S. Sharma, P. Halstenberg, S. K. Gill, A. M. M. Abeykoon, G. Kwon, M. Topsakal, B. Layne, K. Sasaki, Y. Zhang, S. M. Mahurin, S. Dai, C. J. Margulis, E. J. Maginn and V. S. Bryantsev, *Phys. Chem. Chem. Phys.*, 2020, **22**, 22900-22917.
16. S. Roy and V. Bryantsev, *Journal of Physical Chemistry B*, 2018, **122**, 12067-12076.
17. S. Roy, G. Schenter, J. Napoli, M. Baer, T. Markland and C. Mundy, *Journal of Physical Chemistry B*, 2020, **124**, 5665-5675.
